# Separate Introns Gained within Short and Long Soluble Peridinin-Chlorophyll *a*-Protein Genes during Radiation of *Symbiodinium* (Dinophyceae) Clade A and B Lineages

**DOI:** 10.1371/journal.pone.0110608

**Published:** 2014-10-17

**Authors:** Jay R. Reichman, Peter D. Vize

**Affiliations:** 1 US Environmental Protection Agency, Western Ecology Division, Corvallis, Oregon, United States of America; 2 Oregon State University, Department of Botany and Plant Pathology, Corvallis, Oregon, United States of America; 3 University of Calgary, Department of Biological Sciences, Calgary, Alberta, Canada; University of Veterinary Medicine Hanover, Germany

## Abstract

Here we document introns in two *Symbiodinium* clades that were most likely gained following divergence of this genus from other peridinin-containing dinoflagellate lineages. Soluble peridinin-chlorophyll a-proteins (sPCP) occur in short and long forms in different species. Duplication and fusion of short *sPCP* genes produced long *sPCP* genes. All short and long *sPCP* genes characterized to date, including those from free living species and *Symbiodinium sp.* 203 (clade C/type C2) are intronless. However, we observed that long *sPCP* genes from two Caribbean *Symbiodinium* clade B isolates each contained two introns. To test the hypothesis that introns were gained during radiation of clade B, we compared *sPCP* genomic and cDNA sequences from 13 additional distinct Caribbean and Pacific *Symbiodinium* clade A, B, and F isolates. Long *sPCP* genes from all clade B/B1 and B/B19 descendants contain orthologs of both introns. Short *sPCP* genes from *S. pilosum* (A/A2) and *S. muscatinei* (B/B4) plus long *sPCP* genes from *S. microadriaticum* (A/A1) and *S. kawagutii* (F/F1) are intronless. Short *sPCP* genes of *S. microadriaticum* have a third unique intron. *Symbiodinium* clade B long *sPCP* sequences are useful for assessing divergence among B1 and B19 descendants. Phylogenetic analyses of coding sequences from four dinoflagellate orders indicate that introns were gained independently during radiation of *Symbiodinium* clades A and B. Long *sPCP* introns were present in the most recent common ancestor of *Symbiodinium* clade B core types B1 and B19, which apparently diverged sometime during the Miocene. The clade A short *sPCP* intron was either gained by *S. microadriaticum* or possibly by the ancestor of *Symbiodinium* types A/A1, A3, A4 and A5. The timing of short *sPCP* intron gain in *Symbiodinium* clade A is less certain. But, all *sPCP* introns were gained after fusion of ancestral short *sPCP* genes, which we confirm as occurring once in dinoflagellate evolution.

## Introduction

Spliceosomal introns are present in all known eukaryotic genomes, yet the density of introns is highly variable across lineages [1]. The consensus view is that the variations result from dynamic and lineage specific gains and losses of spliceosomal introns within eukaryotic genomes over the course of their evolution [2]–[7]. This paper focuses on introns of dinoflagellates. Apparently, early chromalveolate genomes (those from apicomplexans, ciliates, cryptomonads, dinoflagellates, haptophytes and heterokonts) were intron-rich before their descendant lineages experienced net intron losses. There are indications that this was especially true for the Alveolate ancestors of dinoflagellates [8]. Until recently introns had only been observed in a few dinoflagellate protein coding genes [9]–[14]. A draft genome for *Symbiodium minutum* (clade B) has now identified several more genes in this species with introns [15]. However, investigations so far have not determined when any dinoflagellate introns were inserted. Here we characterize previously unknown introns within genes coding for water soluble peridinin-chlorophyll *a*-proteins (sPCP) that are unique to photosynthetic peridinin-containing dinoflagellates. Our major objective is to estimate when the *sPCP* introns that we discovered in *Symbiodinium* clade A and B isolates were gained relative to other events in dinoflagellate evolution, which are described below.

Peridinin-containing dinoflagellate species are distributed across the Gonyaulacales, Gymnodiniales, Peridiniales, Prorocentrales and Suessiales, which contains the genus *Symbiodinium*. The Suessiales apparently diverged from the Gymnodiniales [16], and this split may have occurred before or during the Paleocene (66–56 MYA) [17]. *Symbiodinium* species are the dominant endosymbionts of marine invertebrates, particularly within shallow tropical oligotrophic waters where coral reefs exist. Over the last four decades, morphological, biochemical and molecular data have shown that the genus *Symbiodinium* is a remarkably diverse assemblage of taxa with variation of ecological zonation, phylogeographic distribution, physiological adaptation and host association [18]–[29]. Hosts include corals, anemones, jellyfish, sponges, zooanthids, bivalves, and forams (reviewed by Coffroth and Santos [30]). There are now nine recognized clades within *Symbiodinium*, designated A–I [18], [23], [25], [31]–[34]. A consensus cladogram of the relationships among *Symbiodinium* clades is shown in the Figure S1. As molecular markers improved, detection of genetic diversification within *Symbiodinium* has progressed from generic to population and clone levels [30], [35]–[38]. The *Symbiodinium* intraclade “types” referred to in the current work are *sensu* previous reports [23], [24]. Our notation (e.g. B/B1/B184) follows the convention of clade/nuclear rDNA ITS type/chloroplast rDNA type (where published data is available). Radiations within clades A and B are of particular relevance to the *sPCP* intron findings presented in this paper. The basal clade A is a cluster of forms that have radiated from two ancestral types, A/A1 and A/A3; both of which have been found in the Caribbean and Indo/Pacific [39]. Evidence suggests that radiation of clade A began during the Oligocene (33.9 to 23 MYA) [17]. The clade B lineage shares dominance in the Caribbean with clade C [26], [40]–[42]. B/B1 and B/B19 are the ancestral core types that formed the base of the Caribbean radiation of clade B that began near the Pliocene/Pleistocene transition (4 to 2 MYA) during which the isthmus of Central America closed [43]. Representatives of B/B1 and B/B19 are also present in the Indo-Pacific indicating that these independent lineages diverged before the Atlantic and Pacific basins separated. The split between B/B1 and B/B19 is thought to have occurred sometime during the Miocene (23 to 5.3 MYA) [17], [43]. Despite the documented diversification of lineages within *Symbiodinium*, relatively few species have been formally named in this genus since the original description of *S. microadriaticum* (A/A1) [44]. However, two new *Symbiodinium* species have been described recently; *S. minutum* and *S. psygmophilum* that are B/B1 and B/B2 (descendant of B/B19) types respectively [45]. Coincidently, strains of *S. microadriaticum*, *S. minutum* and *S. psygmophilum* are among the isolates sampled here that were found to contain *sPCP* introns. Phylogenies of clade A and B taxa based on molecular data from previous publications are presented in the following: [38], [39], [43].

Background on sPCP structure and *sPCP* gene family evolution provide important context for the sequence and phylogenetic positions of *sPCP* intron gain events. Peridinin-containing dinoflagellates including *Symbiodinium* taxa express species-specific, functionally similar isoforms of short (15–17 kD dimeric) and/or long (32–35 kD monomeric) sPCPs [46]–[52]; see Figure S2. Soluble PCPs are nuclear-encoded, plastid-directed, light-harvesting antennae [53]–[59]. Soluble PCP transcripts can be highly expressed and have been among the most frequently recovered EST clones from *Lingulodinium polyedra* [59] and *Symbiodinium* sp. CassKB8 ( = *S. microadriaticum*) [60]. The signal and transit peptides that route both short and long sPCP apoproteins into chloroplasts have amino acid sequences that are similar to those found on several other nuclear-encoded plastid-directed proteins [54]–[56], [58]. However, previous studies found that mature sPCP peptides do not share significant amino acid sequence homology with other known chromophore-binding proteins (≤36% identity) [53], [61], including phycobiliproteins or dinoflagellate membrane-bound, light-harvesting complexes (known as acpCP and iPCP) that contain chlorophyll *a*, chlorophyll *c_2_* plus peridinin [12], [62], [63]. Mature long sPCP apoproteins contain paired domains that do resemble each other and short sPCPs. The similarity of N-terminal and C-terminal domains of long sPCPs is the result of a pseudo-axis of symmetry in their amino acid sequences, which indicates that dinoflagellate long *sPCP* genes arose via duplication and fusion of short *sPCP* genes [53],[54],[56],[64]. This hypothesis was supported by Weis et al. [65]. Their report characterized the short *sPCP* cDNA from *S. muscatinei* (B/B4) and compared overlapping regions of short and long predicted sPCP amino acid sequences from two *Symbiodinium* and three free-living species. The authors concluded that there was an ancient branching within the dinoflagellate *sPCP* gene family leading to separate short and long *sPCP* clades. Today, there is a seemingly random phylogenetic assortment of peridinin-containing dinoflagellates expressing short, long or both size classes of sPCPs [23], [46], [65], [66]. The ecological and evolutionary reasons for the distribution remain obscure but it is possible that fusion of short *sPCP* genes happened more than once. Another point regarding *sPCP* gene evolution is that both classes of apoproteins are encoded by tandem arrays of nuclear genes that vary in terms of copy number and heterogeneity across dinoflagellate lineages. Apparently, variation within *sPCP* gene families is the primary source of sPCP isoform diversity [54]–[57].

All short and long *sPCP* genes characterized to date have been intronless. These genomic and cDNA sequences have only come from *Amphidinium carterae*, *Heterocapsa pygmaea*, *Lingulodinium polyedra* ( = *Gonyaulax polyedra*) and *Symbiodinium sp.* 203 (C/C2) [54]–[57]. In contrast, we first observed that long *sPCP* genes from distinct *Symbiodinium* clade B isolates from the Caribbean scleractinian coral species *Dichocoenia stokesii* and *Diploria strigosa* each contained two spliceosomal introns. This prompted us to ask, were these introns recent evolutionary additions to *Symbiodinium* clade B or actually ancient features that were lost from other peridinin-containing dinoflagellates? In response, this paper addresses several issues regarding the newly discovered *sPCP* introns. To test the initial hypothesis that introns were gained during radiation of clade B, here we compare *sPCP* genomic and cDNA sequences from an additional 13 genetically distinct Caribbean and Pacific *Symbiodinium* clade A, B, and F isolates. Those characterizations reveal a third unique intron in the short *sPCP* genes of *S. microadriaticum*. We assess the radiation of intron-containing long *sPCP* cassettes among clade B isolates. Next, this report presents phylogenetic analyses on both size classes of *sPCP* coding sequences from free-living and *Symbiodinium* dinoflagellates to evaluate the evolutionary distribution of introns. We consider alternative explanations for the distribution data; intron gain during radiation of *Symbiodinium* lineages vs. loss of ancient introns that could have been present before divergence of the Suessiales. We show similarity between exon junction sequences found in clade A and B isolates and potential exon boundaries [67]–[72] within intronless *sPCP* genes of other dinoflagellates. We also compare sPCP predicted amino acid sequences across five genera for two purposes; 1) to reevaluate the Weis et al. [65] conclusion that long *sPCP* genes originated from an ancient single fusion of duplicated short *sPCP* genes, and if supported; 2) to further gauge the timing of when the three introns were acquired relative to the duplication-fusion event of *sPCP* genes in ancient peridinin-containing dinoflagellates.

## Results

### Introns found in long *sPCP* genes in Caribbean *Symbiodinium* clade B isolates

Our *Symbiodinium* isolates from Bahamian host coral colonies of *Dichocoenia stokesii* and *Diploria strigosa* are hereafter referred to as Dstok28 and Dstrig102 respectively. Nuclear large subunit rDNA sequences from Dstok28 and Dstrig102 were used for preliminary identification of these samples, both of which most closely matched *Symbiodinium* clade B accessions in GenBank (data not shown). Outward facing U448/L423 *sPCP* primers [57] produced 2.3 kb amplicons from Dstok28. Although direct sequences of these products became unresolved after short read lengths, BLAST search results from the 5′ and 3′ flanks indicated that they were long *sPCP* CDS. The Dstok28 U448/L423 products were then cloned and sequenced by primer walking. As anticipated, the U448/L423 primers amplified across the *sPCP* intergenic spacer between tandem coding regions. The 5′ half of the downstream partial CDS was interrupted by two introns in all clones. Novel primers that flanked the Dstok28 *sPCP* coding region amplified ∼1.9 kb products that contained 1.1 kb complete coding sequences from Dstok28 and Dstrig102. The CDS of both isolates coded for long form sPCPs. Sequences of *sPCP* cassettes from Dstok28 were distinct from Dstrig102 with an average percent similarity of 93.4 for paired comparisons (accessions JN602521-JN602528 vs. JN60251N602520). The average similarity between coding sequences within these cassettes was 96.6, therefore, 3.2% dissimilarity came from changes in non-coding regions of the cassettes. The remaining 3.4% originated in the coding sequences, which had an estimated non-synonymo7-Jus to synonymous substitution ratio (dn/ds) of 0.14. Interestingly, *sPCP* coding sequences of both isolates were arranged as three exons separated by two introns in the same positions (described below). These observations prompted us to form the hypothesis that introns were gained in long *sPCP* genes during radiation of *Symbiodinium* clade B. We also recognized the need for more accurate determination of the relationships among Dstok28, Dstrig102 and other clade B taxa in conjunction with genomic and cDNA *sPCP* sequence data from additional *Symbiodinium* isolates across clades.

**Figure 1:**
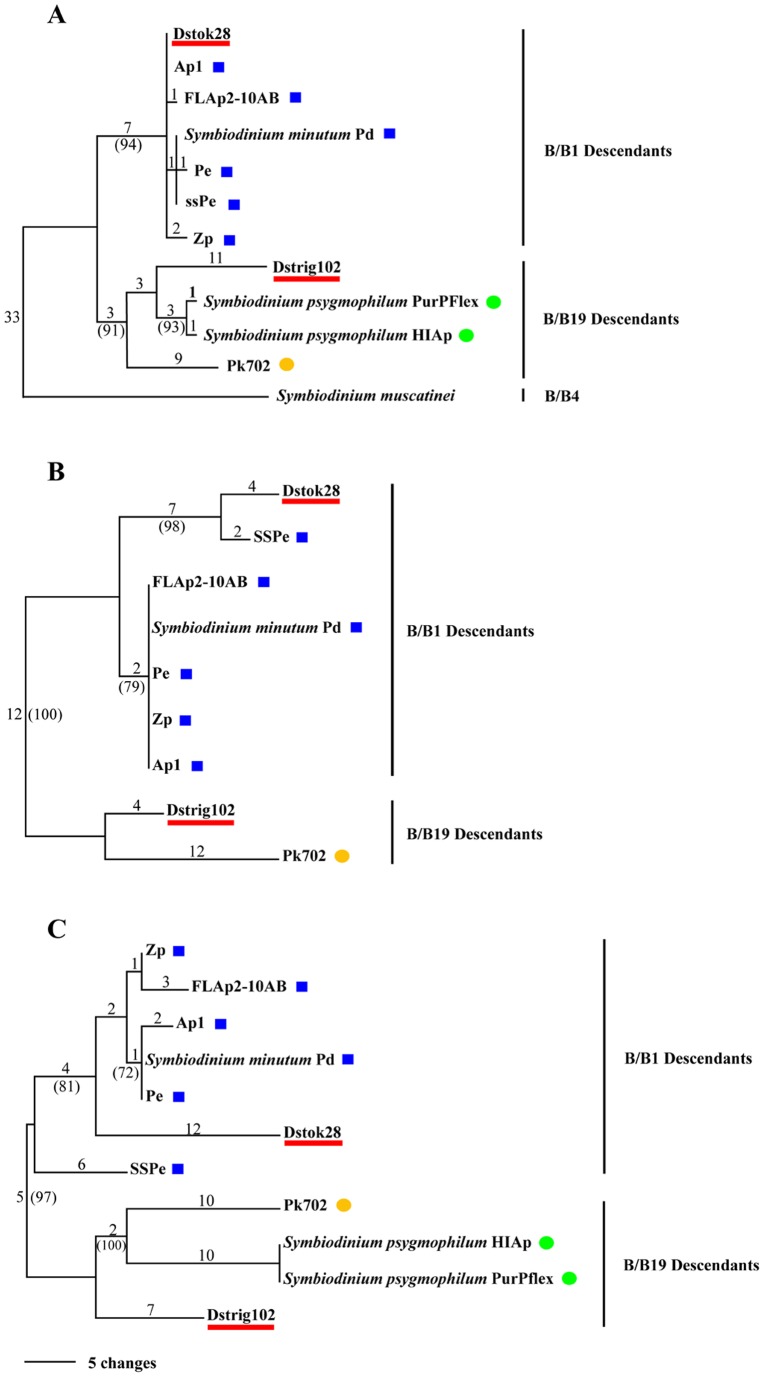
Molecular identification of *Symbiodinium* spp. Dstok28 and Dstrig102 isolates. The most parsimonious trees are shown for the (A) ITS1-ITS2, (B) CA4.86 and (C) Si15 data matrices of direct sequences. Branch lengths are shown above the branches. Support values >70% based on 1000 bootstrap trees are shown in parentheses below branches. Blue squares = B/B1/B184; Green circles = B/B2/B224 (descendant of B/B19); Orange circles = B/B19/B211. All three markers indicate that Dstok28 and Dstrig102 (underlined in red) are B/B1 and B/B19 descendants respectively.

At the second tier of molecular identification, separate comparisons of sequence data from ITS1-ITS2, CA4.86 and Si15 loci from Dstok28, Dstrig102 and ten known *Symbiodinium* clade B isolates (Table S1) produced phylogenetic trees with generally similar topologies (Figure 1). The CA4.86 locus was null for *S. muscatinei*, HIAp and PurPflex strains of *S. psygmophilum.* Si15 was also null for *S. muscatinei*. Nevertheless for all three markers, Dstok28 and Dstrig102 were placed within highly supported subclades of B/B1 and B/B19 descendants respectively. Also, Dstok28 and Dstrig102 were found to be genetically distinct from each other and from all other clade B isolates sampled here.

### Two introns are conserved among clade B long *sPCPs* and a third unique intron is located in clade A short *sPCP* genes

**Figure 2:**
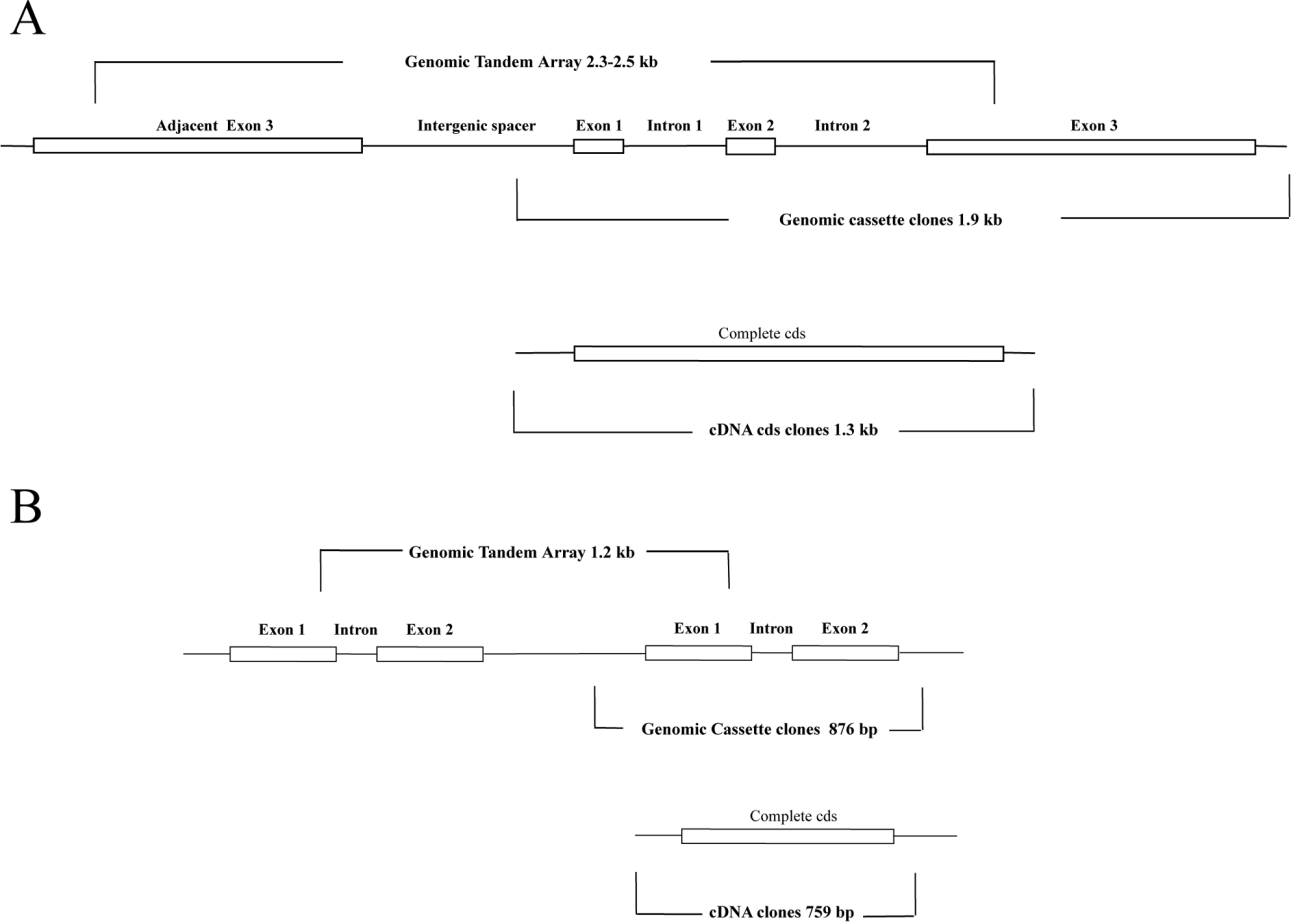
Maps showing the organization of *Symbiodinium sPCP* tandem genes containing introns and the corresponding cDNAs. (A) Long *sPCP* genes from clade B/B1 and B19 descendant isolates contained two introns. The CDS identified in genomic and cDNA clones was ∼1.1 kb. (B) Short *sPCP* genes from *S. microadriaticum* (clade A/A1) had one intron and the CDS was ∼600 bp.

To test the hypothesis that *sPCP* introns observed in Dstok28 and Dstrig102 were gained during radiation of clade B, we started by comparing *sPCP* genomic and cDNA sequences from an additional 13 genetically distinct Caribbean and Pacific *Symbiodinium* clade A, B, and F isolates (Table S1). In all clade B/B1 and B/B19 descendants, two distinct introns were spliced into phase one sites of conserved glycine codons within long *sPCP* genes. Short *sPCP* genes from *S. pilosum* (A/A2) and *S. muscatinei* (B/B4) plus long *sPCP* genes from *S. microadriaticum* (A/A1) and *S. kawagutii* (F/F1) were intronless. However, when sequences of *S. microadriaticum* short *sPCP* ∼876 bp genomic cassettes and ∼759 bp cDNAs of were aligned, a third intron spliced into a phase zero site of an alanine codon was found (see Figure 2 maps). The long *sPCP* intron 1 splice site occurred at a glycine codon between the predicted cleavage sites that demark the signal peptide, transit peptide and mature peptide. The intron 2 splice site glycine codon was located in amino acid position 33 of the long sPCP mature peptide. The *S. microadriaticum* intron splice site falls between codons for the lysine and alanine in amino acid positions 42 and 43 of the short sPCP mature peptide.

The observation of a *sPCP* intron outside of clade B raised the possibility that our initial hypothesis was incorrect, and indicated that further analyses were required. Figure S3 contains detailed maps that summarize the organization of all new genomic and cDNA *sPCP* clone sets and relative locations of primers. Also, subalignments of genomic and cDNA sequences that we used to identify the splice sites for the three introns described here are shown in Figure S4. The subalignments include amino acids predicted from both genomic and cDNA sequences. The five predicted amino acids upstream and downstream of each clade B intron insertion point were conserved across the isolates sampled. All three introns have different, yet conserved, donor (exon|intron) and acceptor (intron|exon) site boundaries. The non-canonical *Symbiodinium* boundaries (5′ splice site MWG|gy and 3′ splice site sag|GY consensus across *sPCP* introns) are consistent with the findings of Bachvaroff and Place [13] and Shoguchi et al. [15] for other dinoflagellate introns. We also observed that the long *sPCP* CDS consensus contained (AAG)(GCC) codons at positions 42 and 43 plus (AAA)(GCC) at positions 205 and 206 of the mature polypeptide. These pairs of adjacent lysine and alanine codons were in the same relative positions on opposite sides of the pseudo-axis of symmetry. Furthermore, their sequences correspond to the intron splice site location of the *S. microadriaticum* short *sPCP* genes. But as described above, the clade B long *sPCP* introns that we found were not inserted between either of these pairs of codons. Instead, the clade B introns were both inserted further upstream in the CDS.

Table 1 shows size ranges for features of *Symbiodinium sPCP* genomic cassettes with introns and corresponding cDNAs. The data revealed conserved patterns of organization for the long *sPCP* genes of the clade B isolates. Exon 1 occurred in discrete size classes of 121, 124, 127 and 130 bp. The variation in exon 1 resulted from indels of whole codons within the CDS. The vast majority of these indels changed the number of tandem alanine codons in the signal peptide. While polymerase slippage cannot be excluded as a source of this variation, there was no evidence of such sequencing errors at other simple sequence repeats within the cassettes. In contrast, exons 2 and 3 were consistently 126 bp and 845 bp each. Differences of length between complete coding sequences of genomic and cDNA clones were predominantly due to indels of alanine codons in the region described above. Introns 1 and 2 tended to have continuous size variations; 209–264 bp and 297–412 bp. Notable exceptions to this trend were B/B2/B224 *S. psygmophilum* strains HIAp and PurPflex that had introns 1 (228 bp) and intron 2 (297 bp) with fixed sizes. Short *sPCP* intron sequences of *S. microadriaticum* were substantially smaller than those of clade B intron 1, exons 1 and 2 of the *S. microadriaticum* were only found to be 282 bp and 330 bp.

**Table 1:** Sizes of *Symbiodinium sPCP* exons, introns, genomic CDS and cDNA CDS (bp).

Isolate	Exon 1	Intron 1	Exon 2	Intron 2	Exon 3	Genomic cds	cDNA cds
***Symbiodinium*** ** Clade B long ** ***sPCP***							
Dstok28	121, 124, 127	230–247	126	305–412	845	1092, 1095, 1098	(not available)
Dstrig102	127	209, 212	126	367, 368	845	1098	(not available)
Ap1	124, 127	242–248	126	397–403	845	1095, 1098	1095, 1098
FLAp2 10AB	124, 127	247–254	126	393–408	845	1095, 1098	1095, 1098
Pe	124, 127	233–259	126	305–400	845	1095, 1098	1095, 1098
Pk702	127, 130	212–242	126	359–372	845	1098, 1101	1098
*S. minutum* Pd	127	243–248	126	397, 398	845	1098	1095, 1098, 1101
*S. psygmophilum* HIAp	127	228	126	297	845	1098	1098
*S. psygmophilum* PurPflex	127	228	126	297	845	1098	1098, 1101
SSPe	127	243–264	126	387–398	845	1098	1095, 1098
Zp	124, 127	242–247	126	306–402	845	1095, 1098	1095, 1098
***Symbiodinium*** ** Clade A short ** ***sPCP***							
*S. microadriaticum*	282	108–117	330			612	612

### Divergence of clade B long *sPCP* genomic cassette sequences compared to other loci

**Figure 3:**
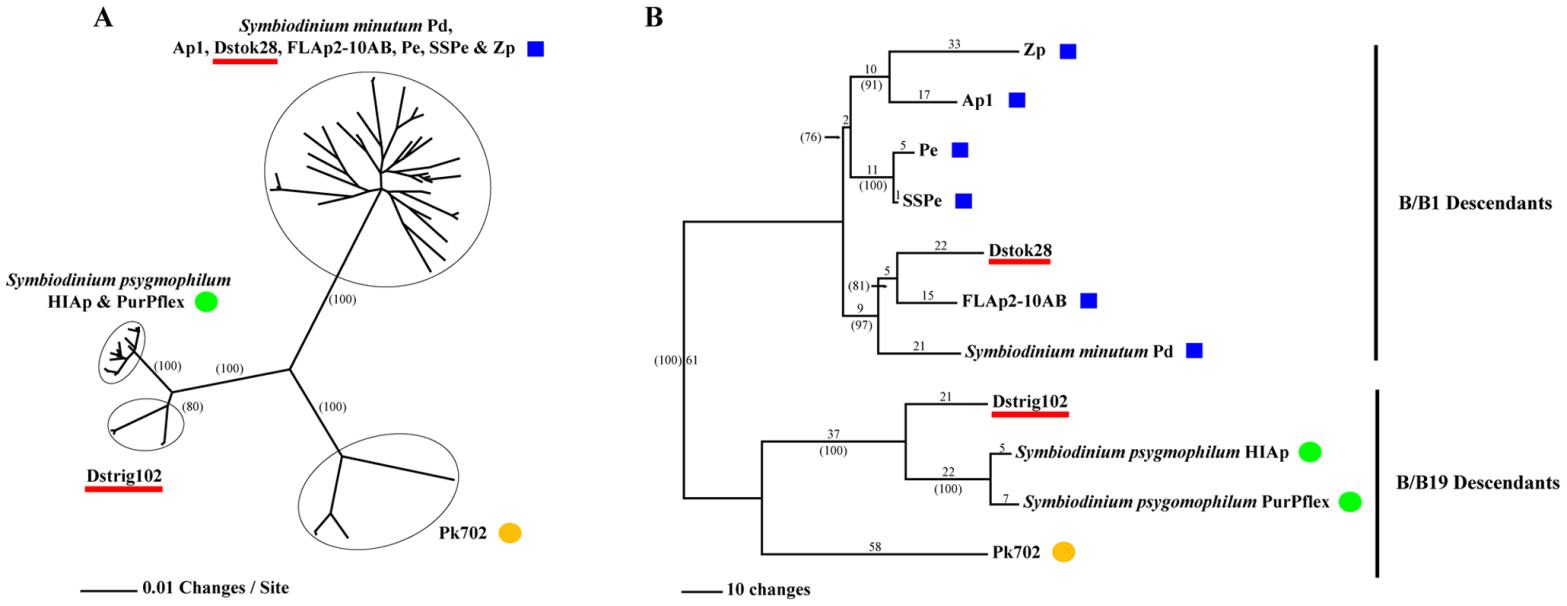
Phylogenies of *Symbiodinium* clade B long *sPCP* cassette sequences. (A) Maximum likelihood tree of all non-chimeric clones. (B) Most parsimonious tree of most frequently recovered non-chimeric clones. Branch lengths for the most parsimonious tree are shown above the branches. Support values >70% based on +1000 bootstrap trees are shown in parentheses. Support values for minor branches of the maximum likelihood tree are not displayed. Blue squares = B/B1/B184; Green circles = B/B2/B224 (descendant of B/B19); Orange circles = B/B19/B211. The positions of Dstok28 and Dstrig102 are underlined in red. The topologies of both trees are congruent with each other.

Phylogenetic trees of clade B long *sPCP* gene sequences (including all exons and introns) are presented in Figure 3. Maximum likelihood relationships among all 138 non-chimeric clones were inferred with SATé (Figure 3A). Subclades containing sequences from B/B1/B184, B/B19/B211 and B/B2/B224 types were resolved from each other with 100% bootstrap support in each case. Branches for individual Dstok28 clones were interdigitated among those from B/B1/B184 isolates Ap1, FLAp2-10AB, Pe, *S. minutum* Pd, SSPe and Zp. A Dstrig102 subclade with 80% bootstrap support was placed as sister to a comingled group of *S. psygmophilum* HIAp and *S. psygmophilum* PurPflex (B/B2/B224) sequences.

A single most parsimonious tree based on analyses of the most frequently recovered clones from each isolate listed above was identified with PAUP (Figure 3B). There was topological agreement between the parsimony and maximum likelihood trees with regard to the highly supported separations among long *sPCP* sequences from B/B1/B184, B/B19/B211 and B/B2/B224 types. As in our ITS1-ITS2, CA4.86 and Si15 phylogenies, *sPCP* sequences from Dstok28 were closely related to known B/B1/B184 taxa. Likewise, Dstrig102 was found to be most similar to, but distinct from B/B2/B224 types. However, the most parsimonious *sPCP* tree showed greater resolution (higher bootstrap support and longer branches) among clade B isolates than trees from the three other loci. This was especially the case for the B/B1/B184 subclade where various isolates had indistinguishable ITS1-ITS2, CA4.86 or Si15 sequences. We also independently estimated the fit of sequence evolution models for our ITS1-ITS2, CA4.86, Si15 and long *sPCP* data, and a different model was found to be optimal for each matrix. The respective models with the best likelihood ratio scores were HKY85 [73], F81 [74], K80 and K80+ G [75]. A comparison of average pairwise distances for each matrix under all four models is presented in Table 2. Under each model, the average distances between long *sPCP* sequences were greater than those for the ITS1-ITS2, CA4.86 or Si15 matrices.

**Table 2:** Average pair-wise distances of *Symbiodinium* Clade B nuclear ITS1-5.8S-ITS2, CA4.86, Si15 and long *sPCP* sequences.

Model	ITS (611 bp)	CA4.86 (227 bp)	Si15 (269 bp)	*sPCP* (1933 bp)
F81	0.01124	0.03629	0.0387	0.04138
K80	0.01125	0.03633	0.03883	0.0415
HKY85	0.01126	0.03638	0.03887	0.04151
K80+ G	0.1205	0.04229	0.4579	0.04834

### *sPCP* coding sequence phylogenies

**Figure 4:**
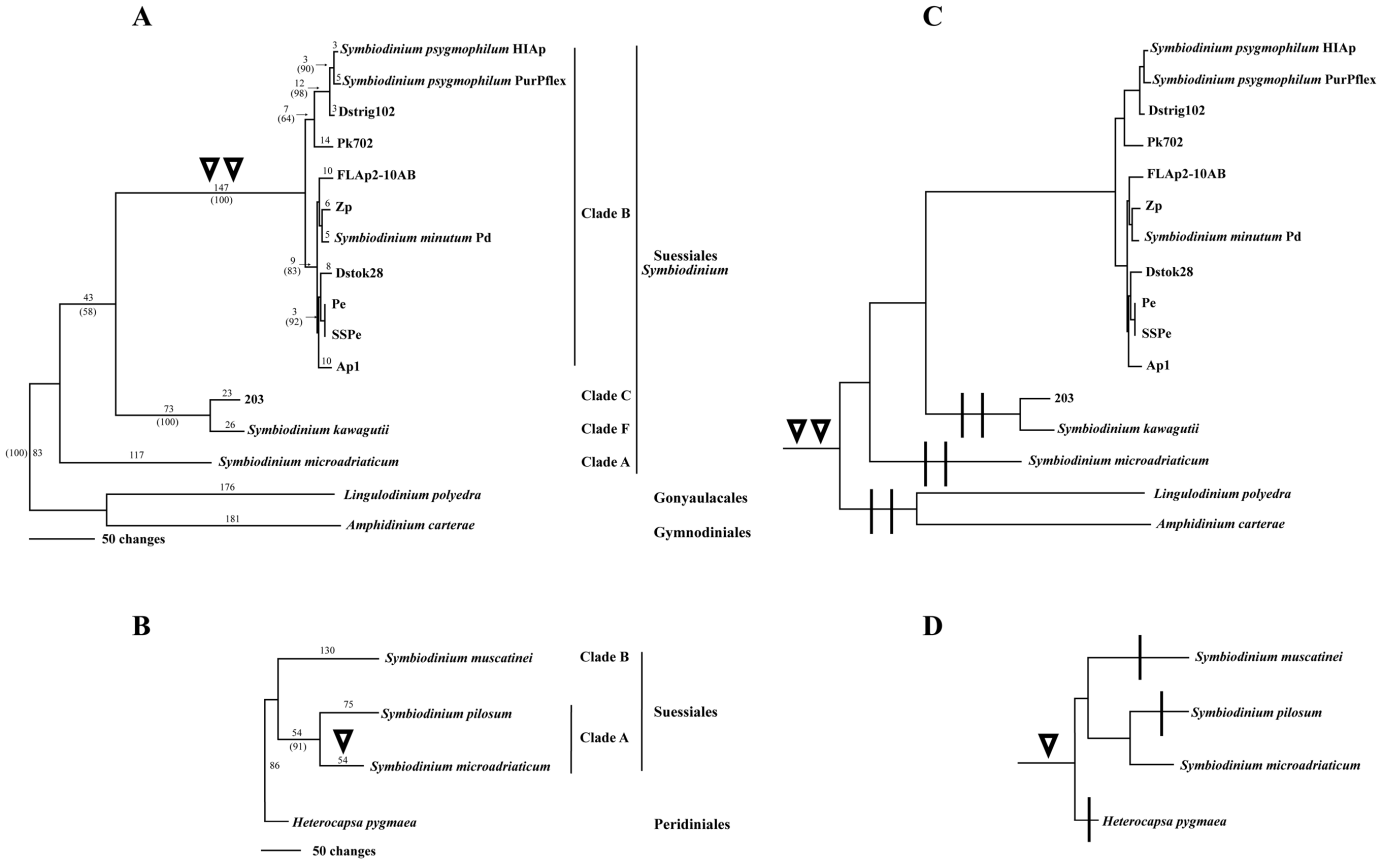
Phylogenies of long and short *sPCP* coding sequences (excluding introns) from four dinoflagellate orders. (A) Most parsimonious tree for long *sPCP* CDS data from the Suessiales, Gonyaulacales and Gynnodiniales. (B) Most parsimonious tree for short *sPCP* CDS from the Suessiales and Peridiniales. Branch lengths are shown above the branches. Support values >50% based on +1000 bootstrap trees are shown in parentheses below branches. Double inverted triangles = the long *sPCP* branch leading to *Symbiodinium* clade B sequences, each of which contained two introns. Single inverted triangle = the short *sPCP* branch for the *S. microadriaticum* sequence that had a single intron. (C) Long *sPCP* tree redrawn to indicate possibility of ancient introns present in the most recent common ancestor of Suessiales, Gonyaulacales and Gynnodiniales. Vertical bar** = **loss of an intron on an ascending branch required to explain the data. (D) Short *sPCP* tree redrawn to indicate possibility of an ancient intron present in the most recent common ancestor of Suessiales and Peridinales. Required losses on ascending branches are indicated as before.

Figures 4A and B respectively show separate most parsimonious trees for long *sPCP* and short *sPCP* coding sequences from four peridinin-containing dinoflagellate orders. Sequences from our most frequently recovered *sPCP* clones and previously published data were used in these analyses. The *sPCP* introns were excluded in the comparisons, so the differences presented in both trees are strictly due to substitutions and indels within the CDS of the samples. The long *sPCP* tree (Figure 4A) is rooted by an outgroup containing free-living species *Lingulodinium polyedra* (Gonyaulacales) and *Amphidinium carterae* (Gymnodiniales). There was 100% bootstrap support for the separation between the free-living and *Symbiodinium* (Suessiales) *sPCP* gene clusters. Furthermore, the relationships among *Symbiodinium* isolates from clades A, B, C, and F were consistent with previous findings estimated by other molecular markers (see Figure S1).

Sequence data for the analysis of short *sPCP* genes was only available for dinoflagellates from the Peridiniales and Suessiales taxa. The short *sPCP* CDS tree (Figure 4B) was rooted by *Heterocapsa pygmaea* (Peridiniales). Among *Symbiodinium* (Suessiales) samples, there was 91% bootstrap support for a relatively close relationship between *S. microadriaticum* and *S. pilosum*. However, the terminal branches for these two clade A taxa contained 54 and 75 changes respectively. *Symbiodinium muscatinei sPCP* was placed sister to the clade A sequences.

The *sPCP* phylogenies shown in Figures 4A and B are annotated to indicate branches within *Symbiodinium* on which more recent intron gains would have occurred. Alternatively, the hypothetical trees in Figures 4C and D illustrate branches inside and outside of *Symbiodinium* on which losses of ancient introns (present before the divergence of the Suessiales) would be required to explain our data.

### Positions of authentic and potential *sPCP* exon junctions

Subsections of aligned sequences from both *sPCP* CDS matrices are presented in Figures S5A and B. Regions flanking intron insertion sites of the clade B long *sPCP* genes and *S. microadriaticum* short *sPCP* genes are aligned with corresponding segments from intronless taxa. Predicted amino acid sequences for these segments are also shown. Interestingly, each of the authentic *sPCP* exon junctions aligned to potential junctions within intronless coding sequences. However, the authentic junctions were not identical to potential junctions within *sPCP* genes of other *Symbiodinium* taxa. Clade B long *sPCP* intron 2 and the *S. microadriaticum* short *sPCP* intron exon junctions exactly matched aligned sequences from more distantly related dinoflagellates, *A. caterae* and *H. pygmaea* respectively.

### Comparisons of short and long sPCP predicted amino acid sequences across five genera

Short and long form sPCP preprotein sequences were predicted from our novel *sPCP* gene sequences and previously published GenBank accessions listed in Table S1. As described above, these accessions included free-living and *Symbiodinium* isolates. The Figure S6 dotplots illustrate several important points regarding the similarity between example pairs of short and long mature sPCP sequences. As has been previously noted [54], [56], [65], short sPCPs share regions of identity with both of the chromophore binding domains of long sPCPs. Just as the short sPCP of *H. pygmaea* is more similar to the C-terminal domain of the *L. polyedra*, the short sPCP of *S. microadriaticum* is most homologous to the C-terminal domain of its own long sPCP. In fact the *H. pygmaea* vs. *L. polyedra* sPCP alignment produced a diagonal with maximum similarity index (SI) of 65.2 over 69 amino acids. However, the corresponding diagonal for short vs. long *S. microadriaticum* sPCPs only had a maximum SI of 53.6 with a length of 56 amino acids. This is the first comparison of short and long form sPCP sequences from the same dinoflagellate isolate. By contrast, when the short sPCPs of *S. microadriaticum* and *H. pygmaea* were aligned, they had an SI maximum of 72.2 across all 151 amino acids. Furthermore, the short sPCP of *S. microadriaticum* and *S. muscatinei* were found to be 80.1 percent identical throughout their entire length.

**Figure 5:**
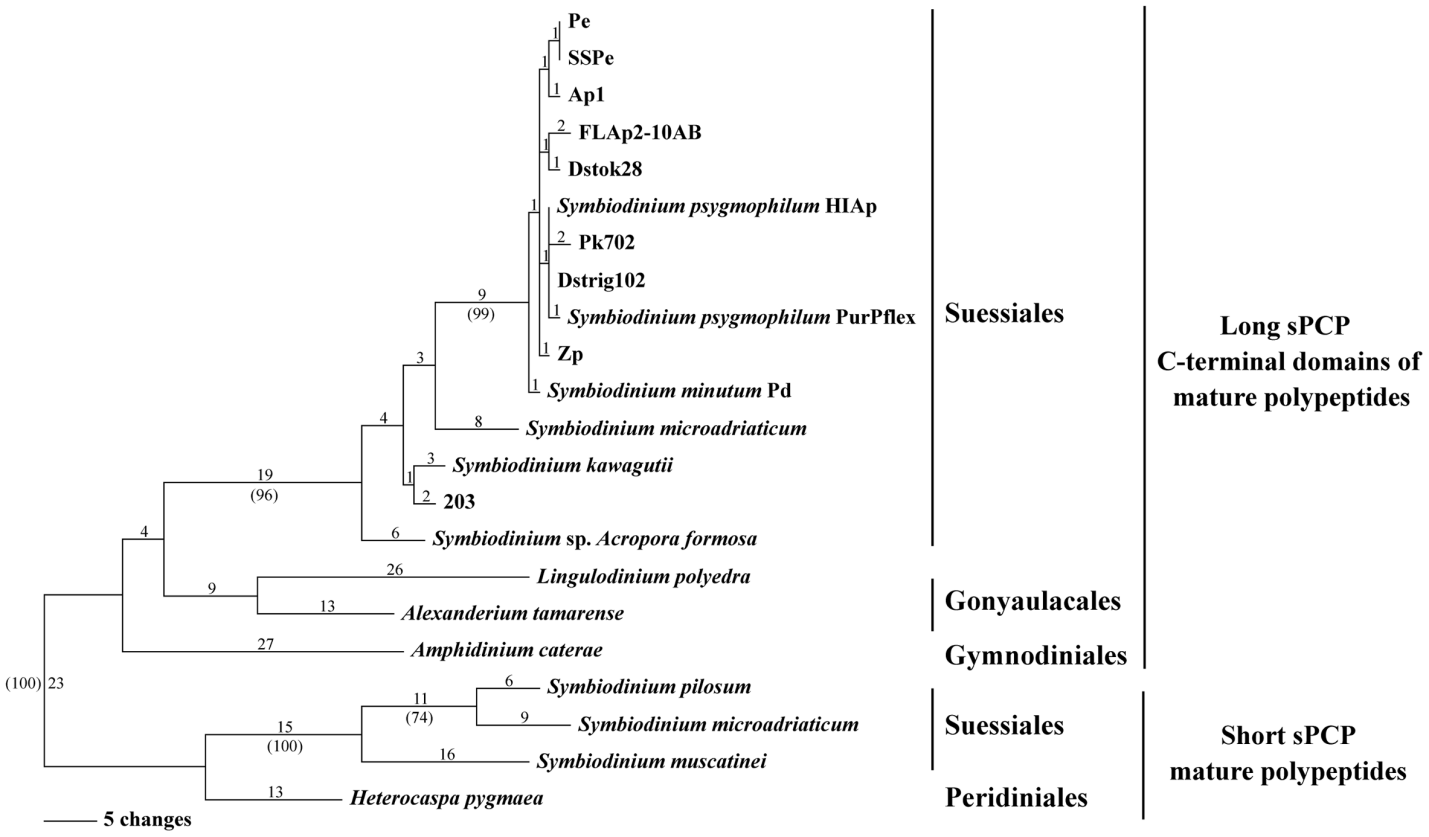
Phylogeny of short and long sPCP predicted amino acid sequences from four dinoflagellate orders. The most parsimonious tree shows unambiguous resolution of short and long *sPCP* sister clades. Branch lengths are shown above the branches. Support values >70% based on +1000 bootstrap trees are shown in parentheses below branches. The long *sPCP* genes were produced from fusion of duplicated short *sPCP* genes that occurred once early during the evolution of peridinin-containing dinoflagellates. Thereafter, short and long *sPCP* genes and corresponding polypeptides diverged.

Figure 5 presents the single most parsimonious phylogenetic tree from our sPCP comparisons. There was 100% statistical support for short and long sPCP sister clades, each containing sequences from free-living and *Symbiodinium* species. Also, the *S. microadriaticum* short and long sPCP sequences were placed within the corresponding sister clades. All short sPCPs were more closely related to each other than to any long sPCPs, and vice versa. The short and long sPCP clades each contained *Symbiodinium* subclades, which had 100% and 96% bootstrap support respectively. In both cases, the *Symbiodinium* groups were found to have diverged from sPCP of free-living dinoflagellates.

## Discussion

This is the first report that describes intron gain events that occurred in *Symbiodinium,* since the divergence of that genus from other peridinin-containing dinoflagellate lineages. There are prior examples of spliceosomal introns in dinoflagellate genes besides *sPCPs*, [9]–[15]. Dinoflagellate introns tend to be less dense in highly expressed tandem repeat genes than in single copy genes with lower expression [13]. But estimates of when particular introns were inserted into other genes were not made in the earlier studies. Here, our initial discovery of introns in long *sPCP* genes of *Symbiodinium* clade B samples from colonies of Caribbean corals *Dichocoenia stokesii* and *Diploria strigosa* was serendipitous. The introns were observed while we were investigating the intragenomic and intergenomic evolution of *sPCP* gene families that are unique to peridinin-containing dinoflagellates. Up until now, all previously characterized short and long *sPCP* genes from free-living and *Symbiodinium* species were shown to be intronless [54]–[57]. Therefore, *Symbiodinium* clade B long *sPCP* genes with introns appeared strikingly anomalous and pointed to the possibility of a relatively recent intron gain event. At first, the main objective of this study was to test the hypothesis that *sPCP* introns were gained during the radiation of clade B. Once comparisons of genomic and cDNA sequences from several *Symbiodinium* taxa revealed a third unique intron in the short *sPCP* gene of *S. microadriaticum*, this objective was augmented to also test for a separate gain event that occurred at some point during the evolution of clade A.

### Long *sPCP* introns gained prior to split between *Symbiodinium* B/B1 and B/B19

In earlier reports on the diversity and ecology of *Symbiodinium* dinoflagellates, shallow colonies of *D. stokesii* (collected from 1–5 m) contained *Symbiodinium* type B/B1 strains. Colonies of *D. strigosa* from the same basin and depth range were found to have B/B1 or B/B1 plus C1 types, while B/B19/B38 has been found within this coral species below 10 m [38], [42]. Likewise, the Bahamian colonies that we sampled contained similar *Symbiodinium* clade B strains. We used multilocus phylogenetic analyses of ITS1-ITS2, plus CA4.86 and Si15 (a.k.a. B7Sym15) direct sequences to identify our Dstok28 and Dstrig102 samples from these same host species. CA4.86 and Si15 were previously developed as *Symbiodinium* clade B markers [35], [36]. Dstok28 and Dstrig102 sequences were compared to those of ten well characterized *Symbiodinium* clade B cultured isolates from the Caribbean and Pacific. The most parsimonious trees for each marker were congruent in the identification of Dstok28 and Dstrig102 as B/B1 and B/B19 descendants respectively. We found no indication from nuclear large subunit rDNA (data not shown) or ITS1-ITS2 sequences that either of our samples from *D. stokesii* or *D. strigosa* housed other *Symbiodinium* types at the time that the samples were collected. The phylogenetic relationships that we inferred among these two isolates and clade B taxa with known genotypes indicate that Dstok28 is not *S. minutum*, yet it is a member of the B/B1/B184 lineage also associated with corals in the genera *Diporia*, *Colpophyllia*, *Favia*, *Isophyllia*, *Isophylastrea*, and *Orbicella* ( = *Montastraea*) [42]. Dstrig102 was similar to but distinct from B/B19/B211 and B/B2/B224 *S. psygmophilum* isolates studied here. Because ITS2 DGGE fingerprinting [32], [43] and chloroplast large subunit rDNA sequencing [24] were not conducted on the Dstok28 and Dstrig102 isolates, it is possible that the putative type designations that we assigned to them, could still be more accurately determined by these methods.

The fact that both Dstok28 and Dstrig102 were genetically distinct yet each contained two introns in the same locations of their long *sPCP* genes suggested that these introns were present in the most recent common ancestor of the B1 and B19 Caribbean core types. As anticipated, orthologs of these two distinct introns were present in the long *sPCP* genes from all nine of the other *Symbiodinium* clade B/B1 and B/B19 descendant taxa that we sampled. The orthology was established by three criteria; conservation of intron positions within the CDS, splice junction borders, and intron sequences. We note that the synapomorphy of long *sPCP* introns was shared among the sampled clade B isolates that came from very distantly separate geographic locations including, the Bahamas, the Florida Keys, Panama, Hawaii and Japan. Also, we did not find any *Symbiodinium* clade B isolates that contained only long *sPCP* intron 1 or intron 2. Taken together, our results indicate that both long *sPCP* introns were present in the most recent common ancestor of *Symbiodinium* clade B core types B1 and B19. These introns predate the divergence between B1 and B19 and the Caribbean expansion of clade B. Furthermore, both long *sPCP* introns may have been gained near the same point in time during the Miocene by the ancestor to B1 and B19 in what would later become the Pacific basin. The retention of both long *sPCP* introns throughout the radiation of B/B1 and B/B19 descendants suggests that they have not been deleterious and selected against. Whether the introns are selectively neutral or advantageous to clade B strains is not yet known.

### The independent gain of the short *sPCP* intron found in *S. microadriaticum*

*Symbiodinium microadriaticum* was the only isolate in which we found an intron in short *sPCP* genes. The discovery of an intron within the short *sPCP* genes of *S. microadriaticum* raised the possibility that the introns may have been present in short *sPCP* genes prior to their duplication and fusion. However, results of our further analyses indicate that the unique *S. microadriaticum* intron was not orthologous with either of the clade B long *sPCP* introns, and that it was gained independently of those in clade B. The timing of short *sPCP* intron gain found in *Symbiodinium* clade A is less certain than that of the clade B long *sPCP* introns. Although there were no introns found in the *S. pilosum* short *sPCP* genes, it is entirely possible that short *sPCP* introns may be present in other descendants of the ancestor to *Symbiodinium* A/A1, A/A3, A/A4 and/or A/A5 types (see [38]) that were not sampled here. When those determinations are made, it will then become possible to more accurately place the short *sPCP* intron gain event on phylogenies of *Symbiodinium*.

### On dinoflagellate intron splice site variation

The *sPCP* intron 5′ splice site MWG|gy and 3′ splice site sag|GY consensus sequences resemble corresponding MAG|gt and mag|GT sequences of introns in mammals, plants and fungi. Previously, *Crypthecodinium cohnii* was found to have U1, U2, U4, U5 and U6 snRNA homologues [76], [77]. *Symbiodinium minutum* was also shown to have clustered genes for these snRNAs [15]. This suggests that dinoflagellate spliceosomes may be assembled like those of yeast and higher eukaryotes. However, the lack of highly conserved 5′ splice site sequences near dinoflagellate exon|intron boundaries [9]–[15] draws into question which adaptations of splicing machinery dinoflagellates have evolved to recognize and process variable intron junctions. Additional empirical characterizations of dinoflagellate spliceosomal structure and function are needed.

### Utility of the long *sPCP* locus as a phylogenetic marker for *Symbiodinium* Clade B

Soluble peridinin-chlorophyll a-proteins are unique to peridinin-containing dinoflagellates. That suggests that *sPCP* nucleotide and amino acid sequences could be informative and useful for studying the evolution of these ecologically important microalgae. However, the results presented here further demonstrate that *sPCP* gene families have undergone substantial intragenome and intergenome diversification. Consequently, utilization of *sPCP* loci as phylogenetic markers requires two special considerations. First, *sPCP* PCR and sequencing primers tend to have limited reactivity across lineages and gene size classes. The long *sPCP* flanking primers designed here from Dstok28 did amplify cassettes from other B/B1 and B/B19 descendants. As we have shown, the long *sPCP* locus containing two introns appears to be readily useful for assessing divergence among B1 and B19 descendants. These results complement phylogenetic analyses from other loci such as ITS1-ITS2, CA4.86 and Si15. For example, our clade B long *sPCP* phylogenies (Figure 3A and B) are congruent with the *S. minutum* and *S. psygmophilum* species designations recently put forth by LaJeunesse et al. [45]. Furthermore, our results show that Dstok28 is closely related to but distinct from *S. minutum.* On the other hand, the Dstok28 primers did not amplify the long *sPCP* genes of any other *Symbiodinium* isolates that we sampled outside of clade B or the short *sPCP* genes of *S. muscatinei*. Second, amplification of *sPCP* genes from heterogeneous tandem arrays can produce a mixture of amplicons that can confound direct sequencing [57]. Here we have compared the most frequently recovered, non-chimeric clones. Alternatively, it has been suggested that DGGE fingerprints of *sPCP* amplicons may be phylogenetically informative. This approach could also be used to identify the most prevalent sequence within a *sPCP* array (Todd LaJeunesse, *personal communication*).

### Sequence position and phylogenetic distribution of *sPCP* introns

As shown in Figure S4, all long *sPCP* intron 1 s were inserted in a phase one of a glycine codon near the signal peptide-transit peptide cleavage site. The combination of phase and gene position agrees with previous findings on human introns, which have a highly significant excess of phase one introns in signal peptide cleavage site preprotein codons [78]. The authors of that study suggested that amino acid sequences surrounding signal peptide cleavage sites are significantly enriched in phase 1 proto-splice sites, which favor insertion of spliceosomal introns. Likewise, each long *sPCP* intron 2 was also inserted into a phase one of a glycine codon at amino acid position 33 of the mature protein. The insertion of both long *sPCP* introns into phase one positions of glycine codons is consistent with statistical results indicating that phase one introns are most often located in glycine codons [79].

In our long *sPCP* CDS phylogeny (Figure 4A), the well supported cluster of *Symbiodinium* clade B sequences was separated from intronless *sPCP* genes *Symbiodinium* sp. 203 (clade C), *S. kawagutii* (clade F), *S. microadriaticum* (clade A) and those of free-living species *L. polyedra* and *A. caterae* within the outgroup. Our results could be explained by gains of both long *sPCP* introns in the most recent common ancestor of the B1 and B19 descendants. On the other hand, the introns may have been present in long *sPCP* genes of the most recent common ancestor of the Gonyaulacales, Gymnodiniales and Suessiales, though this appears very unlikely. This alternative interpretation of our results would require at least six independent single intron losses across the remainder of the sequences compared here or three events in which *both* introns were lost simultaneously (compare Figures 4A and C). It is most parsimonious to infer the gain of long *sPCP* introns on the branch leading to B1 and B19 sub-lineages.

In contrast to the long *sPCP* introns of *Symbiodinium* clade B, the *S. microadriaticum* short *sPCP* intron was inserted into a phase zero position between lysine and alanine codons (Figure S4). Intron insertions frequently follow AAG lysine codons in primates as well [79]. The presence of a short *sPCP* intron in *S. microadriaticum* but absent in *S. pilosum* (at the base of clade A), *S. muscatinei* and *H. pygmaea* is most simply explained by a gain in *S. microadriaticum* (Figures 4B) or possibly by the ancestor of *Symbiodinium* types A/A1, A3, A4 and A5 (see [38]). If the intron was present in the most recent common ancestor of the Suessiales and Peridiniales, then three losses would be required to explain our results (compare Figures 4B and D).

### Alignment of authentic and potential exon splice junctions

We evaluated our aligned *sPCP* data in light of previous evidence that sequences commonly flanking spliceosomal introns may precede insertion of introns. Insertions (↓) can apparently occur at potential exon splice junction like the MAG↓G proto-splice site motif (M = C or A) and at sequences resembling cryptic splice sites, which are often similar to proto-splice sites. We have shown that the exon boundaries for all three *sPCP* introns align with potential exon junction sequences that are present in intronless *sPCP* genes of taxa in other lineages (Figure S5). Our results suggest that, potential junctions existed before the insertions occurred. The clade B long *sPCP* intron 1 was inserted at the location of potential exon junctions that apparently arose after the divergence of the Suessiales from Gymnodiniales. Potential exon junctions existed much earlier at CDS positions where the long *sPCP* intron 2 and the short *sPCP* intron were inserted. Long *sPCP* intron 2 was inserted at a site already present in the most recent common ancestor of the Suessiales, Gymnodiniales and Gonyaulacales. While a potential exon junction site where the short *sPCP* intron was inserted was present within the most recent common ancestor of the Suessiales and Peridiniales. Base pair differences between aligned authentic and potential exon junctions are primarily due to synonymous substitutions that could have occurred since introns were inserted.

### All three introns were gained after the divergence of short and long *sPCP* genes

Extant peridinin-containing dinoflagellates variously express sPCP preproteins of one or both size classes. Mature short sPCP predicted amino acid sequences can be aligned to either domain of mature long sPCPs of dinoflagellates from other genera. But alignments of the corresponding short and long *sPCP* nucleotides are impeded by numerous substitutions at both synonymous and non-synonymous positions within codons. Here we compared sPCP predicted amino acid sequences across five genera for two purposes; 1) to reevaluate the Weis et al. [65] conclusion that long *sPCP* genes originated from an ancient single fusion of duplicated short *sPCP* genes, and if supported; 2) to further gauge the timing of when the three introns were acquired relative to the duplication-fusion event of *sPCP* genes in ancient peridinin-containing dinoflagellates. We found greater similarity between short sPCP amino acids and the C-terminal domain of long sPCPs, rather than the N-terminal domain. Also, differences between sPCP sequences are dominated by the ancient divergence in short and long *sPCP* gene families. As shown by Weis et al. [65] and extended here, short sPCPs are more similar to each other than to any long sPCPs, and vice versa. A very clear demonstration of this from the current work comes from *S. microadriaticum* which has short sPCPs that are more closely related to *H. pygmaea* than its own long sPCPs (Figure 5 and Figure S6).

Because *S. microadriaticum* contains both short and long *sPCP* genes, it gave us the chance to gauge the short intron gain relative to the duplication and fusion event that produced long *sPCP* genes. Had the short *sPCP* intron in *S. microadriaticum* actually been gained early in dinoflagellate evolution, prior to the duplication and fusion or even prior to *sPCP* gene transfer to the dinoflagellate nuclear genome, then orthologous introns should have been present in the short *sPCP* genes of other dinoflagellates and in both domains of long *sPCP* genes in several extant taxa. We found no evidence for either of these possibilities. To the contrary, each of the three introns was distinct in terms of position within *sPCP* CDS, splice sites and nucleotide sequence. These results indicate that the short *sPCP* intron found in *S. microadriaticum* was gained after the short and long *sPCP* genes diverged.

### On distribution of short and long *sPCP* genes in *Symbiodinium* clades A and B

It is interesting to note that *S. pilosum* and *S. muscatinei* branch at the bases of clades A and B respectively [38], and both have short *sPCP* genes while later descendants in both lineages have short and long form genes or only long. For example, *S. microadriaticum* has short and long *sPCP* genes, and all Bs characterized to date besides *S. muscatinei* have long *sPCP* genes. In light of a single duplication-fusion event, the ancestor of A/A1, A2, A3, A4 and A5 probably had both short and long *sPCP*s. Under that scenario, long *sPCPs* that were lost from A2 after it diverged for other As were retained in A1. Both forms may still be present in A3, A4 and A5, but that has not been determined yet. Likewise, the ancestor of B/B4, B1 and B19 probably had both short and long *sPCP*s. If true, then long *sPCPs* were lost from B4 and short *sPCPs* were lost from the ancestor of B1 and B19. For *Symbiodinium* clades A and B, as with other peridinin-containing dinoflagellates, loss or retention of sPCP genes of a particular size class could have been due to selection or random occurrences. Whether or not there are conditions under which it is advantageous to have short or long *sPCPs* is an important question in dinoflagellate evolution that remains to be answered.

### Conclusion

Here we have presented multiple lines of evidence that *sPCP* introns were gained independently during the radiation of *Symbiodinium* clades A and B. Furthermore, the results indicate that all three *sPCP* introns were gained well after duplication and fusion of ancestral short *sPCP* genes, which we confirm as occurring once early in the evolution of peridinin-containing dinoflagellates.

## Materials and Methods

### Algal Samples

The dinoflagellate isolates, sources and sequence accessions compared in this study are presented in Table S1. *Dichocoenia stokesii* and *Diploria strigosa* coral colonies containing the *Symbiodinium* sp. endosymbiotic dinoflagellates where we first observed *sPCP* introns (Dstok28 and Dstrig102 respectively) were collected from patch reefs near Lee Stocking Island, Bahamas. Coral samples were collected by JRR under CITES permit: Bahamas –97/156). The *D. stokesii* host was located at a depth of 2 m at 23°49'3.12”N; 76°11'17.40”W. The *D. strigosa* colony was also at a depth of 2 m at 23°46'55.10”N; 76° 6′40.60”W. *Symbiodinium* sp. cells were extracted from coral tissue with the use of a WaterPic. The slurries were filtered through gauze to remove coral debris. The captured microalgae were washed with 0.45 µ filtered seawater, centrifuged, and pellets were stored in 96% ethanol. Cells were frozen until DNA extraction. Dr. Virginia Weis donated an anemone (*Anthopleura elegantissima*) collected from the Central Oregon Coast, USA at approximately 44°29'35.87”N; 124° 5′10.22”W from which *Symbiodinium muscatinei* (B/B4) cells were isolated by maceration and centrifugation. No specific permissions were required for these locations/activities. The dinoflagellate cells were washed in 0.45 µ filtered seawater, and then stored on ice until nucleic acids were extracted.

*Symbiodinium* sp. clade B cultures Ap1 (B/B1/B184), FLAp2-10AB (B/B1/B184), HIAp (B/B2/B224), Pd (B/B1/B184), Pe (B/B1/B184), Pk702 (B/B19/B211), PurPflex (B/B2/B224), SSPe (B/B1/B184) and Zp (B/B1/B184) were provided by Dr. Scott Santos. During the course of this study, Pd was identified as a strain of *S. minutum*. Also, HIAp and PurPflex were shown to be strains of *S. psygmophilum* [45]. *Symbiodinium pilosum* (A/A2) was shared by Dr. Mark Warner. *Symbiodinium microadriaticum* (A/A1; isolate 61; CCMP 2464) and *S. kawagutii* (F/F1; isolate 135; CCMP 2468) were obtained from the Provasoli-Guillard National Center for Culture of Marine Phytoplankton. Algal cultures were grown in 0.22 µ filtered seawater enriched with F/2 [80] from Sigma Inc. Cultures were placed under 40W plant and aquarium fluorescent lights at ∼80 µ mole quanta/m^2^/s on 12 h light-12 h dark cycle and were maintained at 25°C.

### Molecular Techniques and Data Collection

Genomic DNA was extracted from dinoflagellate samples with Plant DNeasy Kits (Qiagen, Valencia, CA). DNA was quantified with a NanoDrop spectrophotometer (Thermo). Total RNA was extracted from cultured *Symbiodinium* species by the Trizol protocol and included DNAse I treatment (Invitrogen, Carlsbad, CA). A SuperScript Kit (Invitrogen) was used for reverse transcription. The oligo dT primer from the kit was used for first strand cDNA synthesis from polyadenylated nuclear mRNA. Subsequent amplification of double-stranded cDNA was done with gene specific primer pairs that flanked the CDS as described below.

PCRs were optimized in Mastercycler Gradient thermal cyclers (Eppendorf, Hauppauge, NY). The 25 µl reactions contained 40 ng DNA, 125 µM of each dNTP, 0.4 µM primers, 1× buffer, and 1.25 U *Taq* polymerase (Roche Applied Science, Indianapolis, IN). Oligos were synthesized by Operon (Huntsville, AL). All thermal profiles consisted of an initial 95°C for 3 min, 40 cycles of 95°C for 30 s, annealing temperatures (see citations for existing primers and Figure S3 for novel primers) for 30 s and extensions at 72°C (variable time depending on expected product length), with a final extension of 72°C for 3 min. PCR products were purified with QIAquick Gel Extraction kits (Qiagen) and quantified as above.

Approximately 20 ng of amplicon template was directly sequenced in both directions to minimize confounding issues from recombinant artifacts [81], [82]. Templates were labeled in 10 µl half-reactions using BigDye v3.1 (Applied Biosystems, Carlsbad, CA) chemistry. Labeled fragments were purified with CleanSeq Kits (AgenCourt Bioscience, Beverly, MA). Capillary electrophoresis and data collection occurred on an ABI 3100. When direct sequence reads could not be resolved due to the presence of multiple templates, amplicons were isolated with TA Cloning Kits (Invitrogen) and 20 clones were resequenced from each ligation. Sequence contigs were assembled using Seqman (DNAStar v7-9, Madison,WI). Nucleotide sequences were annotated and translated with SeqBuilder (DNAStar). Signal peptides within predicted sPCP amino acids sequences were identified using the Hidden Markov model (HMM) method of SignalP 3.0 [83]. Novel primers were designed with PrimerSelect (DNAStar). Bellerophon [84] was used to screen out molecular chimeras that may have resulted from PCR amplification of loci in tandem arrays and/or bacterial cloning.

### *Symbiodinium* ITS, CA4.86 and Si15 Phylogenetic Markers

For preliminary clade identification of the Bahamian coral endosymbionts, a fragment of the nuclear lsRNA gene was amplified and sequenced from Dstok28 and Dstrig102 with primers and methods described in [85]. Intraclade relationships among these samples and known *Symbiodinium* clade B isolates (Table S1) were evaluated with sequences from nuclear ITS1-ITS2, CA4.86 and Si15 ( = B7Sym15) loci that were amplified using previously published primers and thermal cycle profiles [35], [36], [86] respectively. PCR products of these loci were directly sequenced.

### Primers for *Symbiodinium sPCP* genomic DNA and cDNA

Novel and previously developed *sPCP* primer sequences, annealing temperatures and maps showing relative primer locations are available in the Figure S3 For all *Symbiodinium* isolates sampled here, direct sequences of *sPCP* products were largely unresolved due do intragenomic variation within tandem arrays. Therefore, amplicons were cloned and sequenced as described above. Outward facing *sPCP* primers U448/L423 designed for *Symbiodinium* sp. 203 [57] successfully amplified across the intergenic spacer between adjacent long *sPCP* cassettes from Dstok28. Flanking consensus sequences were used in succession for primer walking across the U448/L423 clones. Additional primers were thereby designed for amplification and sequencing of entire long *sPCP* genomic CDS and cDNA CDS clones from Dstok28, Dstrig102 and other clade B isolates. The sequence from *S. muscatinei* short *sPCP* cDNA GenBank Accession AF42573 [65] was used to design primers that flanked the genomic CDS for that species. *Symbiodinium muscatinei* primers sPCP-F1 (Weis, *personal communication*) and sPCP-R3 [65] also amplified the 3′ end of the short *sPCP* gene from *S. pilosum*. Outward facing primers designed from the sequence of that fragment, amplified between adjacent *S. pilosum* short *sPCP* coding regions. Sequences from tandem CDS clones were then used for primer walking across the intergenic spacer and afterward entire coding regions. Initial long and short *sPCP* gene primers for *S. microadriaticum* were designed from *Symbiodinium* sp. CassKB8 EST sequence KB8 CE ortholog3 [60] and the consensus of KB8 GenBank EST accessions FE538781, FE539772, FE539102 and FE539773 respectively. A similar primer walking approach as described above was used to design additional oligos for amplifying and sequencing both sizes classes of *S. microadriaticum sPCP* CDS. U(-28)/L(1180) *Symbiodinium* 203 *sPCP* primers [57] amplified the long *sPCP* CDS from *S. kawagutii*. The cross-reactivity of new *sPCP* PCR primer pairs was checked among the *Symbiodinium* species sampled here.

### Sequence Comparisons and Statistics

Genomic, cDNA and amino acid sequences were aligned with the Clustal W algorithm within MegAlign (DNAStar). MegAlign was also used to generate dotplots. Protein coding region alignments were adjusted to conserve codon positions. Ratios of non-synonymous to synonymous substitutions in codons were estimated from outputs of SNAP v1.1.1 [87]. Separate phylogenetic comparisons of nucleotide sequence data matrices for ITS1-ITS2, CA4.86, Si15 and our most frequently recovered *sPCP* clones were executed under the parsimony criterion with PAUP*4.0b10 [88]. In these analyses, gaps were treated as missing data and gap coding was used to account for the presence/absence of indels [89]. Four heuristic tree searches were run using random taxon addition with 1000 replications, TBR branch swapping and DELTRAN character state optimization. Bootstrap support analyses were based on 1000 sequence addition replicates.

In order to compare rates of molecular evolution for ITS, CA4.86, Si15 and long *sPCP* sequences, we used the Santos et al. [24] extension of the approach developed by Kusoff et al. [90]. The best fit for different models of evolution were estimated by likelihood ratio tests with MODELTEST v3.7 [91]. The pair-wise distances for each data matrix were generated using PAUP* 4.0b10, and then the average distances were calculated.

Maximum likelihood relationships among all 138 non-chimeric clade B long *sPCP* genomic clones were also estimated with SATé [92], [93]. Four SATé runs were executed using the Clustal W2 aligner, Muscle merger and RAxML tree estimator. Within each run, the maximum subproblem percentage = 20; the stop rule = 24 hour and the decomposition = centroid. Both the maximum likelihood tree and the corresponding alignment identified by SATé were imported into RAxML 7.2.6 [94] to calculate bootstrap support values. RAxML generated 1000 bootstrap trees using the GTRGAMMA model of rate heterogeneity with the shape parameter, GTR rate, and empirical base frequencies estimated from the data matrix.

Potential exon junctions were identified within new and previously published intronless *sPCP* sequences in the same CDS location (positions −3 to +1 relative to intron insertion “↓”) as authentic exon boundaries characterized here. Aligned four base-pair sequences were scored as potential exon junction sequences if they matched exon junctions that we observed in *sPCP* genes, the classic MAG↓G proto-splice site motif [67], or documented cryptic splice site variants [70], [72], [95]. The motifs of potential exon junction sequences from intronless *sPCP* genes were compared to cryptic splice site sequences in the DBASS database of new exon boundaries induced by pathogenic mutations in human disease genes (http://www.dbass.org.uk/). There are no *sPCP* homologues within DBASS, so the position of an identical cryptic splice site sequence in a human gene was not used as a criterion within our scoring rubric for potential *sPCP* exon junctions. The database was only used to check for sequence matches to known cryptic splice sites.

The matrix of predicted amino acid sequences used for our phylogenetic analyses contained short sPCP mature proteins that optimally aligned with the C-terminal domains of long sPCP mature proteins. The sPCP sequences were predicted from genomic and cDNA accessions for all isolates shown in Table S1. As in our *sPCP* gene comparisons above, we restricted novel sPCP sequences to those predicted from our most frequently recovered clones. Gaps were coded for presence/absence, then parsimony analyses and bootstrap support were conducted in PAUP* with settings used in the analyses of the nucleotide data.

## Supporting Information

Figure S1 **Consensus cladogram drawn to emphasize** ***Symbiodinium*** **clades based on molecular data from previous publications** [17], [25], [30], [34], [96] **.**
(TIF)

Figure S2 **Theoretical (A) Short (15 kD) sPCP dimers from** ***Symbiodinium pilosum*** **and (B) Long (35 kD) sPCP monomer from** ***S. kawagutii*** **rendered** ***in silico*** **by the authors using** ***Amphidinium carterae*** **sPCP X-ray crystal structure** [64] **as a scaffold.** Peridinin is gold; chlorophyll *a* is green; apoprotein is grey; 2 digactosyl diacyl glycerol is omitted.
(TIF)

Figure S3 **Detailed** ***Symbiodinium*** **species** ***sPCP*** **maps with primer sequences and annealing temperatures. Intron positions are highlighted in red.** (**A**) *Symbiodinium* spp. B/B1/B184, B/B2/B224 and B/B19/B211 long *sPCP* genes. (**B**) *S. muscatinei* B/B4 short *sPCP* genes. (**C**) *S. microadriaticum* A/A1 long *sPCP* genes. (**D**) *S. microadriaticum* A/A1 short *sPCP* genes. (**E**) *S. pilosum* (A/A2) short *sPCP* genes. (**F**). *S. kawagutii* F/F1 long *sPCP* genes.
(PDF)

Figure S4 **Alignments of partial genomic and cDNA** ***sPCP*** **sequences spanning exon junction sequences within** ***Symbiodinium*** **isolates.** Codon positions and predicted amino acids are shown below the alignments. Exons are uppercase, introns are lowercase. Junction sequences at bases −3 to +2 of the exon|intron donor and intron|exon acceptor termini are in bold.>>> = omitted sequence. (**A**) Clade B long *sPCP* intron 1 sequences at a phase one position of a glycine codon downstream of an alanine codon (G**CT**)(**G↓G**T). (**B**) Clade B long *sPCP* intron 2 sequences at a phase one position of a glycine codon downstream of a proline codon (C**CA**)(**G**↓**G**C). (**C**) *Symbiodinium microadriaticum* short *sPCP* intron sequence at a phase 0 position between lysine and alanine codons (**AAG**)↓(**G**CC).
(PDF)

Figure S5 **(A**) Authentic exon splice junction sequences for *Symbiodinium* clade B long *sPCP* genes align with potential exon splice junctions in other Suessiales, Gonyaulacales and Gynnodiniales taxa. (**B**) Likewise, the authentic exon splice junction sequence for *Symbiodinium microadriaticum* short *sPCP* genes aligns with potential exon splice junctions in other isolates from the Suessiales and Peridiniales. Authentic exon splice junction sequences are bold and enclosed within rectangles. Intron insertion positions are marked with “↓”. Potential exon splice junction sequences are bold with double underlines for exact matches to authentic junctions or single line for match to other splice site motifs.
(TIF)

Figure S6 **Dotplots that compare predicted short and long sPCP mature apoprotein amino acid sequences.** (**A)** The short sPCP of *Heterocapsa pygmaea* is more similar to the downstream domain of the long sPCP from *Lingulodinium polyedra* than (**B)** the short sPCP of *Symbiodinium microadriaticum* is to the downstream domain of the *S. microadriaticum* long sPCP. (**C)** The short sPCP of *S. microadriaticum* is similar throughout its length to the short sPCPs from *H. pygmaea* and (**D)** *S. muscatinei*.
(TIF)

Table S1 **Dinoflagellate taxa and GenBank accessions used for comparison in this study.** Footnote citations for accessions from previous publications are as follows: 1 [23], 2 [24], 3 [65], 4 [97], 5 [40], 6 [98], 7 [57], 8 [53], 9 [54], 10 [55], 11 [56].
(PDF)
