# Correction: Separate Introns Gained within Short and Long Soluble Peridinin-Chlorophyll *a*-Protein Genes during Radiation of *Symbiodinium* (Dinophyceae) Clade A and B Lineages

**DOI:** 10.1371/journal.pone.0117735

**Published:** 2015-01-21

**Authors:** The PLOS ONE Staff

In the first paragraph of the “Results,” the word ‘non-synonymo7-Jus’ should appear as ‘non-synonymous.’

The “Acknowledgements” are missing from the published article. Please view them here.

## Acknowledgments

We thank the following individuals for helpful advice and Symbiodinium isolates; Dr. Scott Santos, Auburn University; Dr. Todd LaJeunesse, Pennsylvania State University; Dr. Mark Warner, University of Delaware; Dr. Virginia Weis and Angela Poole, Oregon State University; and Dr. Robert Trench (ret), UC Santa Barbara. Dinoflagellate cultures were also purchased from the Provasoli-Guillard National Center for Marine Algae and Microbiota (formerly the Center for Culture of Marine Phytoplankton). We also thank Dr. Nicholas Dibb, Imperial College of London for discussion on exon junction sequences. Coral samples were collected by JRR under CITES permit: Bahamas—97/156

The research described herein was developed by the lead author, an employee of the U.S. Environmental Protection Agency (EPA), on his own time. It was conducted independent of EPA employment and has not been subjected to the Agency’s peer and administrative review. Therefore, the conclusions and opinions drawn are solely those of the author and are not necessarily the views of the Agency.
