# Supplementary figures and images for: Separate Introns Gained within Short and Long Soluble Peridinin-Chlorophyll a-Protein Genes during Radiation of Symbiodinium (Dinophyceae) Clade A and B Lineages
Source: PLoS One. 2014 Oct 17;9(10):e110608. doi: 10.1371/journal.pone.0110608 (PMC4201569; doi:10.1371/journal.pone.0110608)

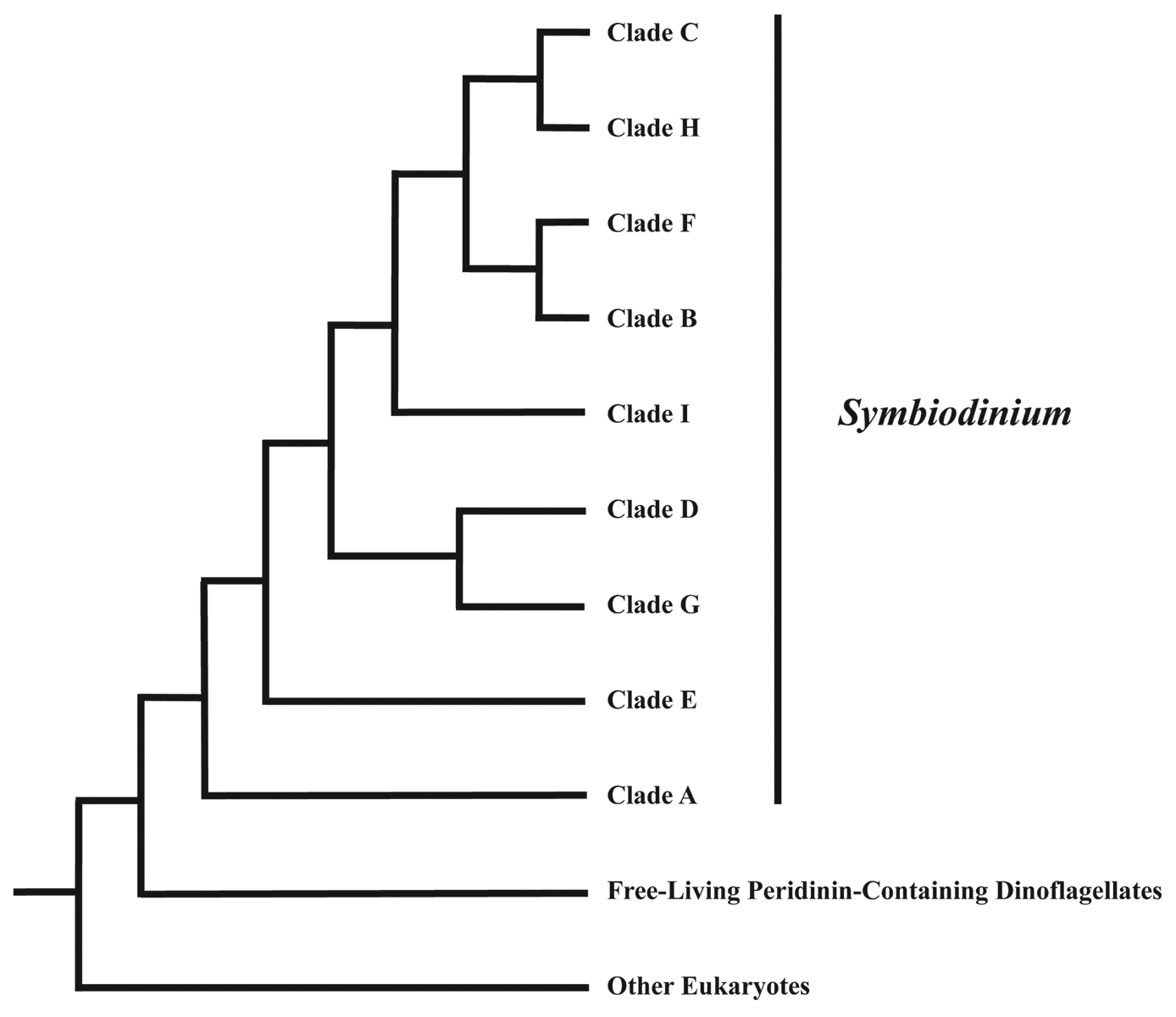

Supplement: Figure S1 — Consensus cladogram drawn to emphasize Symbiodinium clades based on molecular data from previous publications [17], [25], [30], [34], [96] . (TIF) [file pone.0110608.s001.tif]

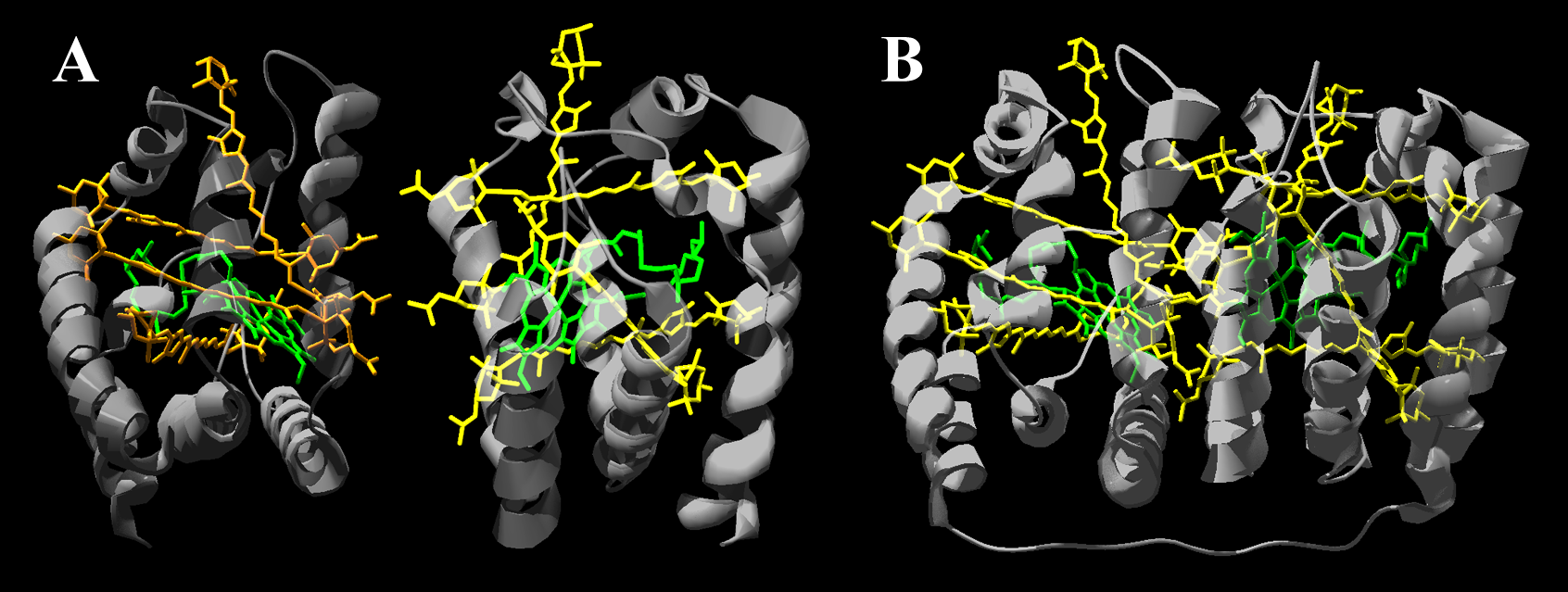

Supplement: Figure S2 — Theoretical (A) Short (15 kD) sPCP dimers from Symbiodinium pilosum and (B) Long (35 kD) sPCP monomer from S. kawagutii rendered in silico by the authors using Amphidinium carterae sPCP X-ray crystal structure [64] as a scaffold. Peridinin is gold; chlorophyll a is green; apoprotein is grey; 2 digactosyl diacyl glycerol is omitted. (TIF) [file pone.0110608.s002.tif]

A

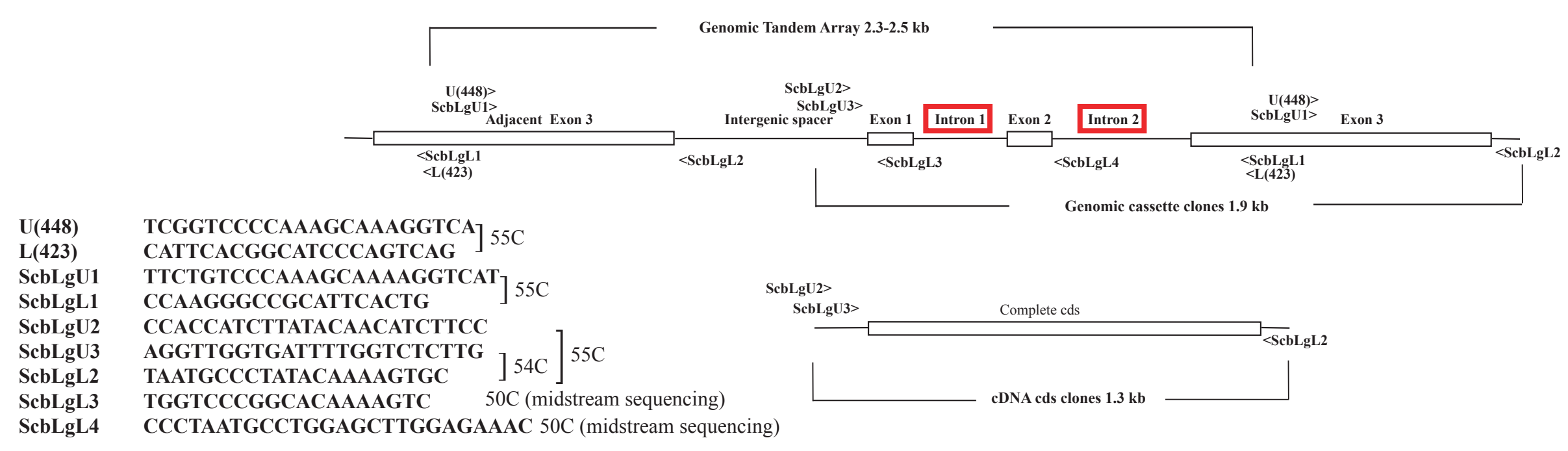

Supplement: Figure S3 — Detailed Symbiodinium species sPCP maps with primer sequences and annealing temperatures. Intron positions are highlighted in red. (A) Symbiodinium spp. B/B1/B184, B/B2/B224 and B/B19/B211 long sPCP genes. (B) S. muscatinei B/B4 short sPCP genes. (C) S. microadriaticum A/A1 long sPCP genes. (D) S. microadriaticum A/A1 short sPCP genes. (E) S. pilosum (A/A2) short sPCP genes. (F). S. kawagutii F/F1 long sPCP genes. (PDF) [file pone.0110608.s003.pdf]

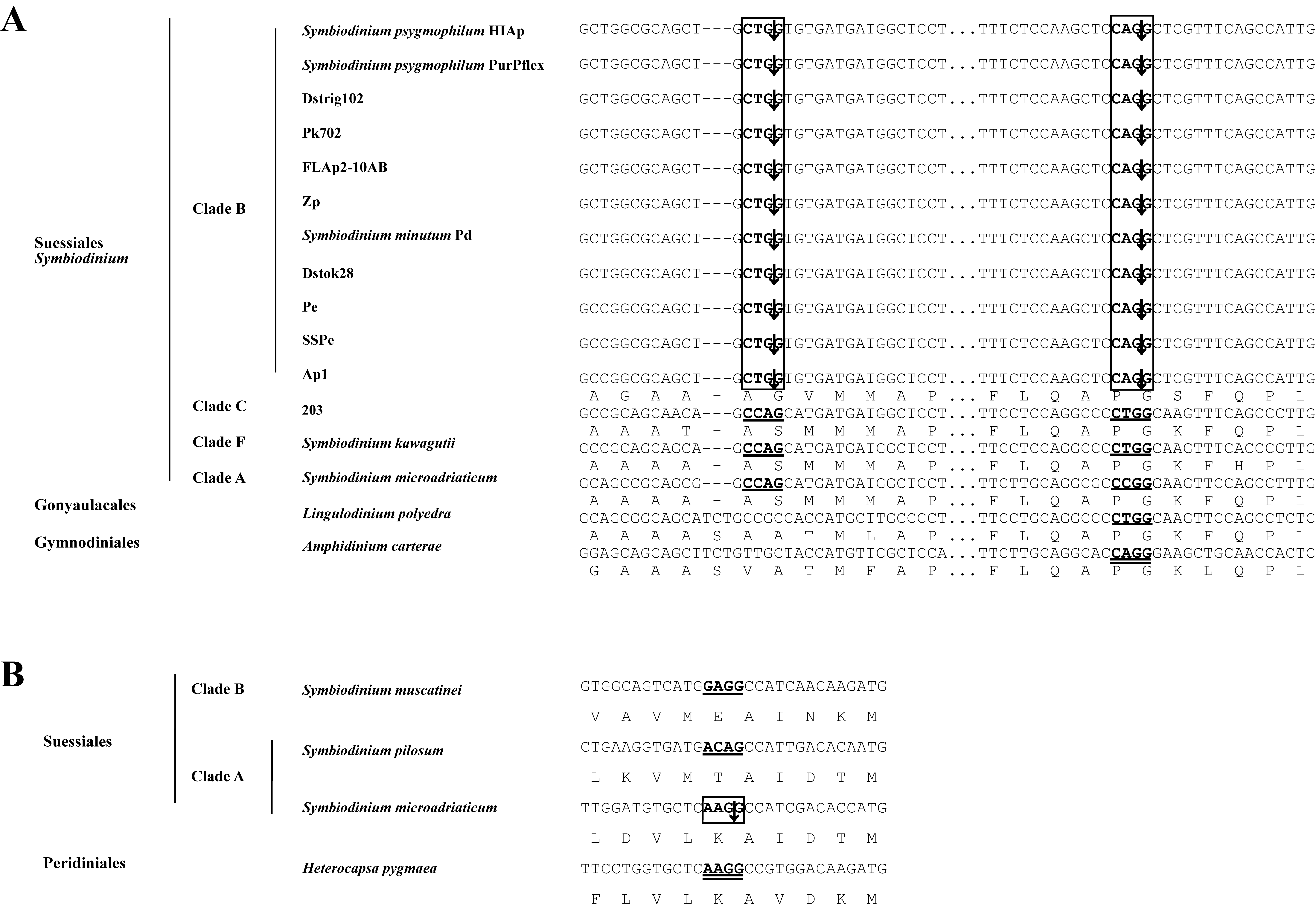

Supplement: Figure S5 — (A) Authentic exon splice junction sequences for Symbiodinium clade B long sPCP genes align with potential exon splice junctions in other Suessiales, Gonyaulacales and Gynnodiniales taxa. (B) Likewise, the authentic exon splice junction sequence for Symbiodinium microadriaticum short sPCP genes aligns with potential exon splice junctions in other isolates from the Suessiales and Peridiniales. Authentic exon splice junction sequences are bold and enclosed within rectangles. Intron insertion positions are marked with “↓”. Potential exon splice junction sequences are bold with double underlines for exact matches to authentic junctions or single line for match to other splice site motifs. (TIF) [file pone.0110608.s005.tif]

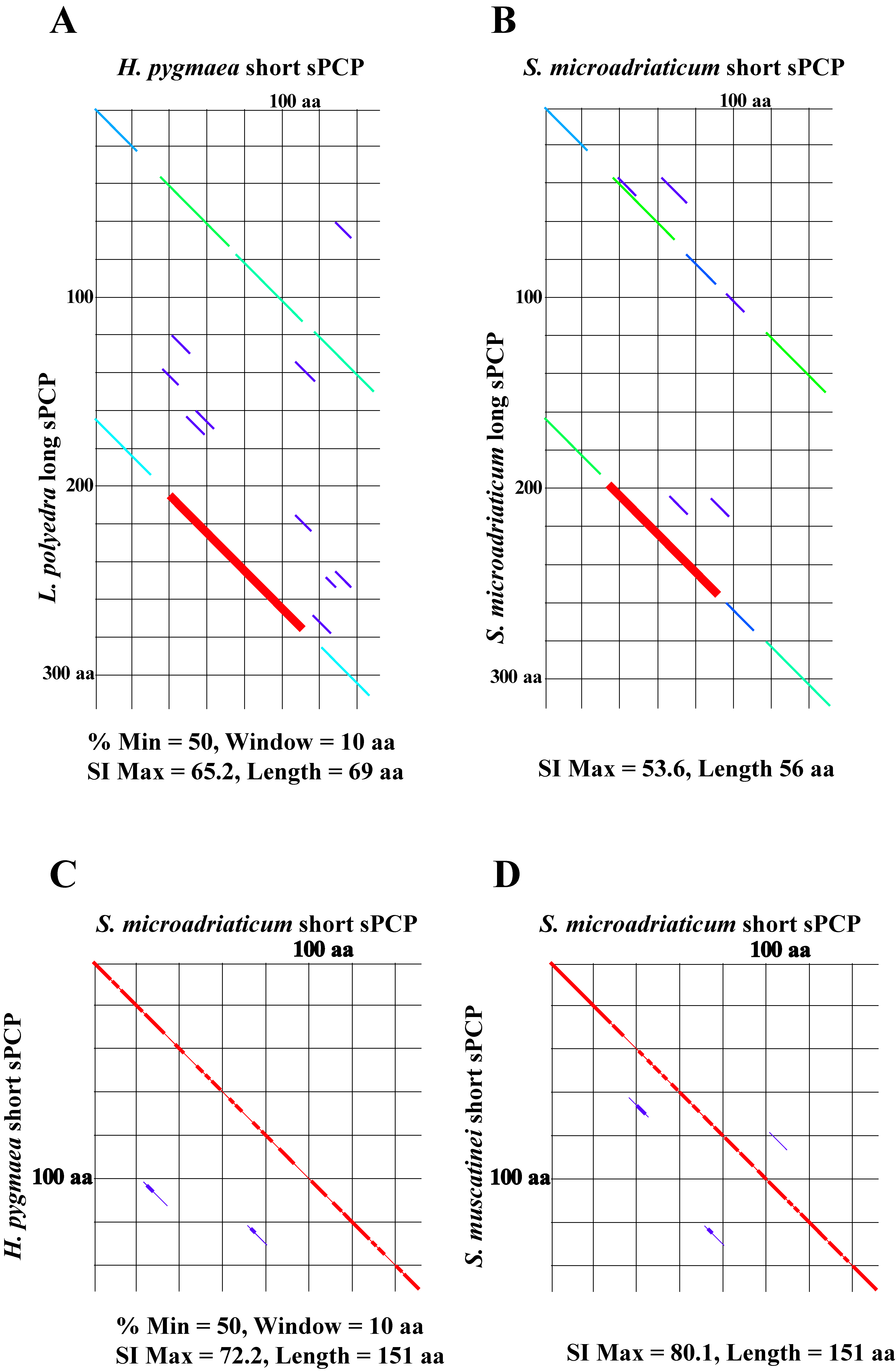

Supplement: Figure S6 — Dotplots that compare predicted short and long sPCP mature apoprotein amino acid sequences. (A) The short sPCP of Heterocapsa pygmaea is more similar to the downstream domain of the long sPCP from Lingulodinium polyedra than (B) the short sPCP of Symbiodinium microadriaticum is to the downstream domain of the S. microadriaticum long sPCP. (C) The short sPCP of S. microadriaticum is similar throughout its length to the short sPCPs from H. pygmaea and (D) S. muscatinei. (TIF) [file pone.0110608.s006.tif]
