# Supplementary material for: Separate Introns Gained within Short and Long Soluble Peridinin-Chlorophyll a-Protein Genes during Radiation of Symbiodinium (Dinophyceae) Clade A and B Lineages
Source: PLoS One. 2014 Oct 17;9(10):e110608. doi: 10.1371/journal.pone.0110608 (PMC4201569; doi:10.1371/journal.pone.0110608)
Supplement: Figure S4 — Alignments of partial genomic and cDNA sPCP sequences spanning exon junction sequences within Symbiodinium isolates. Codon positions and predicted amino acids are shown below the alignments. Exons are uppercase, introns are lowercase. Junction sequences at bases −3 to +2 of the exon|intron donor and intron|exon acceptor termini are in bold.>>> = omitted sequence. (A) Clade B long sPCP intron 1 sequences at a phase one position of a glycine codon downstream of an alanine codon (GCT)(G↓GT). (B) Clade B long sPCP intron 2 sequences at a phase one position of a glycine codon downstream of a proline codon (CCA)(G↓GC). (C) Symbiodinium microadriaticum short sPCP intron sequence at a phase 0 position between lysine and alanine codons (AAG)↓(GCC). (PDF) [file pone.0110608.s004.pdf]

A

```
Dstok28 genomic                             GCTGGCGCAGCTGCTGgtacctaggttcttgt>>>aa.tgttg....tcagGTGTGATGATGGCT
Dstrig102 genomic                           GCCGGCGCAGCTGCTGgtacctaggttcttgt>>>aa.tgttggcagtcagGTGTGATGATGGCT
Ap1 genomic                                 GCTGGCGCAGCTGCTGgtacctaggttcttgt>>>aaatgttggcagtcagGTGTGATGATGGCT
FLAp2-10AB genomic                          GCTGGCGCAGCTGCTGgtacctaggttcttgt>>>aaatgttggcagtaagGTGTGATGATGGCT
Pe genomic                                  GCTGGCGCAGCTGCTGgtacctaggttcttgt>>>aa.tgttggcagtcagGTGTGATGATGGCT
Pk702 genomic                               GCCGGCGCAGCTGCTGgtatctaggttcttgt>>>aa.tgttggcagtcagGTGTGATGATGGCT
SSPe genomic                                GCTGGCGCAGCTGCTGgtacctaggttcttgt>>>aa.tgttggcagtcagGTGTGATGATGGCT
Symbiodinium minutum Pd genomic             GCTGGCGCAGCTGCTGgtacctaggttcttgt>>>aaatgttggcagtcagGTGTGATGATGGCT
Symbiodinium psygmophilum HIAp genomic      GCCGGCGCAGCTGCTGgtacctaggttcttgt>>>aa.tgttggcagtcagGTGTGATGATGGCT
Symbiodinium psygmophilum PurPFlex genomic  GCCGGCGCAGCTGCTGgtacctaggctcttgt>>>aa.tgttggcagtcagGTGTGATGATGGCT
Zp genomic                                  GCTGGCGCAGCTGCTGgtacctaggttcttgt>>>aa.tgttggcagtcagGTGTGATGATGGCT
Ap1 cDNA                                    GCTGGCGCAGCTGCTG................>>>................GTGTGATGATGGCT
FLAp2-10AB cDNA                             GCTGGCGCAGCTGCTG................>>>................GTGTGATGATGGCT
Pe cDNA                                     GCTGGCGCAGCTGCTG................>>>................GTGTGATGATGGCT
Pk702 cDNA                                  GCCGGCGCAGCTGCTG................>>>................GTGTGATGATGGCT
SSPe cDNA                                   GCTGGTGCAGCTGCTG................>>>................GTGTGATGATGGCT
Symbiodinium minutum Pd cDNA                GCTGGCGCAGCTGCTG................>>>................GTGTGATGATGGCT
Symbiodinium psygmophilum HIAp cDNA         GCCGGCGCAGCTGCTG................>>>................GTGTGATGATGGCT
Symbiodinium psygmophilum PurPFlex cDNA     GCCGGTGCAGCTGCTG................>>>................GTGTGATGATGGCT
Zp cDNA                                     GCTGGTGCAGCTGCTG................>>>................GTGTGATGATGGCT
Codon Position                              1231231231231231                                   23123123123123
                                             |  |  |  |  |                                     |  |  |  |  |
Amino acids                                  A  G  A  A  A                                     G  V  M  M  A
```

B

```
Dstok28 genomic                             TTTCTCCAAGCTCCAGgcattagggcattggg>>>gttgcctgttggtgagGCTCGTTTCAGCCA
Dstrig102 genomic                           TTTCTCCAAGCTCCAGgcattagggcattagg>>>gttacctgttgccgagGCTCGTTTCAGCCA
Ap1 genomic                                 TTTCTCCAAGCTCCAGgcattagggcattggg>>>gttgcctgttggtgagGCTCGTTTCAGCCA
FLAp2-10AB genomic                          TTTCTCCAAGCTCCAGgcattagggcattggg>>>gttgcctgttggtgagGCTCGTTTCAGCCA
Pe genomic                                  TTTCTCCAAGCTCCAGgcattagggcattggg>>>gttgcctgttggtgagGCTCGTTTCAGCCA
Pk702 genomic                               TTTCTCCAAGCTCCAGgcattagggcattagg>>>gttacctgttggtgagGCTCGTTTCAGCCA
SSPe genomic                                TTTCTCCAAGCTCCAGgcattagggcattggg>>>gttgcctgttggtgagGCTCGTTTCAGCCA
Symbiodinium minutum Pd genomic             TTTCTCCAAGCTCCAGgcattagggcattggg>>>gttgcctgttggtgagGCTCGTTTCAGCCA
Symbiodinium psygmophilum HIAp genomic      TTTCTCCAAGCTCCAGgcattagggcattagg>>>gttacctgttgccgagGCTCGTTTCAGCCA
Symbiodinium psygmophilum PurPFlex genomic  TTTCTCCAAGCTCCAGgcattagggcattagg>>>gttacctgttgccgagGCTCGTTTCAGCCA
Zp genomic                                  TTTCTCCAAGCTCCAGgcattagggcattggg>>>gttgcctgttggtgagGCTCGTTTCAGCCA
Ap1 cDNA                                    TTTCTCCAAGCTCCAG................>>>................GCTCGTTTCAGCCA
FLAp2-10AB cDNA                             TTTCTCCAAGCTCCAG................>>>................GCTCGTTTCAGCCA
Pe cDNA                                     TTTCTCCAAGCTCCAG................>>>................GCTCGTTTCAGCCA
Pk702 cDNA                                  TTTCTCCAAGCTCCAG................>>>................GCTCGTTTCAGCCA
SSPe cDNA                                   TTTCTCCAAGCTCCAG................>>>................GCTCGTTACAGCCA
Symbiodinium minutum Pd cDNA                TTTCTCCAAGCTCCAG................>>>................GCTCGTTTCAGCCA
Symbiodinium psygmophilum HIAp cDNA         TTTCTCCAAGCTCCAG................>>>................GCTCGTTTCAGCCA
Symbiodinium psygmophilum PurPFlex cDNA     TTTCTCCAAGCTCCAG................>>>................GCTCGTTTCAGCCA
Zp cDNA                                     TTTCTCCAAGCTCCAG................>>>................GCTCGTTTCAGCCA
Codon Position                              1231231231231231                                   23123123123123
                                             |  |  |  |  |                                     |  |  |  |  |
Amino acids                                  F  L  Q  A  P                                     G  S  F  Q  P
```

C

```
Symbiodinium microadriaticum genomic        TTGGATGTGCTCAAGgccattgatccccatt>>>ccgagaacatcgtcagGCCATCGACACCATG
Symbiodinium microadriaticum cDNA           TTGGATGTGCTCAAG................>>>................GCCATCGACACCATG
Codon Position                              123123123123123                                   123123123123123
                                             |  |  |  |  |                                     |  |  |  |  |
Amino acids                                  L  D  V  L  K                                     A  I  D  T  M
```
