# Supplementary material for: Separate Introns Gained within Short and Long Soluble Peridinin-Chlorophyll a-Protein Genes during Radiation of Symbiodinium (Dinophyceae) Clade A and B Lineages
Source: PLoS One. 2014 Oct 17;9(10):e110608. doi: 10.1371/journal.pone.0110608 (PMC4201569; doi:10.1371/journal.pone.0110608)
Supplement: Table S1 — Dinoflagellate taxa and GenBank accessions used for comparison in this study. Footnote citations for accessions from previous publications are as follows: 1 [23], 2 [24], 3 [65], 4 [97], 5 [40], 6 [98], 7 [57], 8 [53], 9 [54], 10 [55], 11 [56]. (PDF) [file pone.0110608.s007.pdf]

Table S1. Dinoflagellate taxa and GenBank accessions used for comparisons in this study

**Isolate**		***Symbiodinium* Clade B Phylogenetic Marker**			**Dinoflagellate *sPCP* Sequences**		
**Suessiales**<br>**(*Symbiodinium* Clade / ITS Type[1]/ cp23s Type[2])**	**Symbiodinium Invertebtate Host &**<br>**Geographical Location**	**ITS1-ITS2**	**CA4.86**	**Si15**	***sPCP* size**	***sPCP* genomic**	***sPCP* cDNA**
Symbiodinium sp. Dstok28<br>(B / B1 / B184)	*Dichocoenia stokesii* (Scleractinaria)<br>Bock Cay, Bahamas	**JN602458**	**JN602470**	**JN602468**	Long	**JN602521-JN602528**	(N/A)
Symbiodinium sp. Dstrig102<br>(B / B19)	*Diploria strigosa* (Scleractinaria)<br>Lee Stocking Island, Bahamas	**JN602456**	**JN602473**	**JN602467**	Long	**JN602517-JN602520**	(N/A)
Symbiodinium muscatinei<br>(B / B4)	*Anthopleura elegantissima* (Actiniaria)<br>Central Oregon Coast, USA	**JN602454**	(null)	(null)	Short	**JN602614-JN602617**	AF42573[3]
Symbiodinium sp. Ap1<br>(B / B1 / B184)	*Aiptasia pulchella* (Actiniaria)<br>Sesoko Jima, Japan	**JN602457**	**JN602474**	**JN602469**	Long	**JN602529, JN602530,**<br>**JN602532, JN602533**	**JN602590-JN602600**
Symbiodinium sp. FLAp2-10AB<br>(B / B1 / B184)	*Aiptasia pallida* (Actiniaria)<br>Long Key, Florida, USA	**JN602455**	JN602472	**JN602466**	Long	**JN602510, JN602512,**<br>**JN602514-JN602516**	**JN602576-JN602589**
Symbiodinium minutum Pd<br>(B / B1 / B184)	*Pocillopora damicornis* (Scleractinaria)<br>Kaneohe Bay, Hawaii, USA	AF360565[4]	AY264305[5]	**JN602464**	Long	**JN602497, JN602498,**<br>**JN602500-JN602502**	**JN602567-JN602573**
Symbiodinium sp. Pe<br>(B / B1 / B184)	*Porites evermanni* (Scleractinaria)<br>Kaneohe Bay, Hawaii, USA	AF360566[4]	**JN602475**	**JN602463**	Long	**JN602494-JN602496,**<br>**JN602499**	**JN602557-JN602566**
Symbiodinium sp. Pk702<br>(B / B19 / B211)	*Plexaura kuna* (Gorgonacea)<br>San Blas, Panama	AF360575[4]	AF474167[6]	**JN602462**	Long	**JN602489,**<br>**JN602491-JN602493**	**JN602601-JN602613**
Symbiodinium psygmophilum HIAp<br>(B / B2 / B224)	*Aiptasia pulchella* (Actiniaria)<br>Kaneohe Bay, Hawaii, USA	AF360564[4]	(null)	**JN602465**	Long	**JN602503-JN602511,**<br>**JN602513**	**JN602574, JN602575**
Symbiodinium psygmophilum PurPflex<br>(B / B2 / B224)	*Plexaura flexuosa* (Gorgonacea)<br>Tennessee Reef, Florida, USA	AF360574[4]	(null)	**JN602461**	Long	**JN602485-JN602488,**<br>**JN602490**	**JN602544-JN602556**
Symbiodinium sp. SSPe<br>(B / B1 / B184)	*Pseudoterogorgia elisabethae* (Gorgonacea)<br>San Salvador, Bahamas	AF360570[4]	AY264307[5]	**JN602460**	Long	**JN602479,**<br>**JN602481-JN602484**	**JN602538-JN602543**
Symbiodinium sp. Zp<br>(B / B1 / B184)	*Zonathus pacificus* (Zoantharia)<br>Kaneohe Bay, Hawaii, USA	**JN602453**	**JN602471**	**JN602459**	Long	**JN602476-JN602478,**<br>**JN602480, JN602531**	**JN602534-JN602537**
Symbiodinium pilosum 185<br>(A / A2)	*Zonathus sociatus* (Zoantharia)<br>Discovery Bay, Jamaica				Short	AY149140-AY149147[7]	**JN602618-JN602624**
Symbiodinium microadriaticum 61<br>(A / A1)	*Cassiopeia xamachana* (Rhizostomeae)<br>Florida Keys, USA				Long<br>Short	**JN602625-JN602628**<br>**JN602634-JN602642**	**JN602629-JN602633**<br>**JN602643-JN602648**
Symbiodinium sp. 203<br>(C / C2)	*Hippopus hippopus* (Bivalia)<br>Republic of Palau				Long	AY149123[7]	
Symbiodinium kawagutii 135<br>(F / F1)	*Montipora capitata* (Scleractinaria)<br>Kaneohe Bay, Hawaii, USA				Long	AY149163-AY149172[7]	**JN602649-JN602659**
Symbiodinium sp.<br>(N/A)	*Acropora formosa* (Scleractinaria)<br>Magnetic Island, Australia				Long		L13613[8]
**Gonyaucales**							
Lingulodinium polyedra	(Free-living)				Long	U93077[9]	
Alexandrium tamarense	(Free-living)				Long		AY847685[10]
**Gymnodiniales**							
Amphidinium caterae	(Free-living)				Long	Z50793[11]	
**Peridiniales**							
Heterocapsa pygmaea	(Free-living)				Short	AJ298193[12]	

1 [23], 2 [24], 3 [65], 4 [97], 5 [40], 6 [98], 7 [57], 8 [53], 9 [54], 10 [55], 11 [56]
